# ArfX2 GTPase Regulates Trafficking From the Trans-Golgi to Lysosomes and Is Necessary for Liver Abscess Formation in the Protozoan Parasite *Entamoeba histolytica*

**DOI:** 10.3389/fcimb.2021.794152

**Published:** 2021-12-17

**Authors:** Yumiko Saito-Nakano, Takashi Makiuchi, Mami Tochikura, Carol A. Gilchrist, William A. Petri, Tomoyoshi Nozaki

**Affiliations:** ^1^ Department of Parasitology, National Institute of Infectious Diseases, Shinjuku-ku, Tokyo, Japan; ^2^ Department of Infectious Diseases, Tokai University School of Medicine, Isehara, Kanagawa, Japan; ^3^ Department of Medicine, University of Virginia, Charlottesville, VA, United States; ^4^ Graduate School of Medicine, The University of Tokyo, Bunkyo, Tokyo, Japan

**Keywords:** *Entamoeba histolytica*, liver abscess, membrane traffic, Arf GTPase, stress resistance

## Abstract

*Entamoeba histolytica* is the causative agent of amoebic dysentery and liver abscess in humans. The parasitic lifestyle and the virulence of the protist require elaborate biological processes, including vesicular traffic and stress management against a variety of reactive oxygen and nitrogen species produced by the host immune response. Although the mechanisms for intracellular traffic of representative virulence factors have been investigated at molecular levels, it remains poorly understood whether and how intracellular traffic is involved in the defense against reactive oxygen and nitrogen species. Here, we demonstrate that EhArfX2, one of the Arf family of GTPases known to be involved in the regulation of vesicular traffic, was identified by comparative transcriptomic analysis of two isogenic strains: an animal-passaged highly virulent HM-1:IMSS Cl6 and *in vitro* maintained attenuated avirulent strain. EhArfX2 was identified as one of the most highly upregulated genes in the highly virulent strain. EhArfX2 was localized to small vesicle-like structures and largely colocalized with the marker for the trans-Golgi network SNARE, EhYkt6, but neither with the endoplasmic reticulum (ER)-resident chaperon, EhBip, nor the cis-Golgi SNARE, EhSed5, and Golgi-luminal galactosyl transferase, EhGalT. Expression of the dominant-active mutant form of EhArfX2 caused an increase in the number of lysosomes, while expression of the dominant-negative mutant led to a defect in lysosome formation and cysteine protease transport to lysosomes. Expression of the dominant-negative mutant in the virulent *E. histolytica* strain caused a reduction of the size of liver abscesses in a hamster model. This defect in liver abscess formation was likely at least partially attributed to reduced resistance to nitrosative, but not oxidative stress *in vitro*. These results showed that the EhArfX2-mediated traffic is necessary for the nitrosative stress response and virulence in the host.

## Introduction

*Entamoeba histolytica* is the etiological agent of human amoebiasis, which presents clinical manifestations including colitis, dysentery, and hepatic liver abscess (Stanley, 2003; Santi-Rocca et al., 2009). Invasion of the intestinal mucosa by the trophozoites leads to colitis and dysentery. In 5%–10% of the symptomatic intestinal infections, the trophozoites are disseminated through the portal vein to other organs including the liver. The survival of trophozoites in the tissues and subsequent formation of niche for parasitism require mechanisms for inactivation of or evasion from the hostile host environments including the immune response.

Multiple cellular mechanisms such as motility, adherence, and cytolysis are coordinately involved in the pathogenesis of *E. histolytica* (Faust and Guillen, 2012). Adherence to the host cells is primarily mediated by 260-kDa Gal/GalNAc lectin on the plasma membrane, which is a heterodimer of 170-kDa heavy and 35/31-kDa light subunits (Petri et al., 2002). The lysine- and glutamic acid-rich protein (KERP1) was initially discovered by the proteome analysis of brush-border binding proteins (Perdomo et al., 2016). KERP1 is implicated in the virulence because KERP1 is involved in the binding to epithelial cells, and its gene expression is upregulated during liver abscess development (Santi-Rocca et al., 2008) and shown to be necessary for the progression of liver abscess (Seigneur et al., 2005). The traffic of KERP1 remains largely elusive because it was shown to be transported extracellularly *via* the non-classical endoplasmic reticulum (ER)–Golgi-independent pathway (Perdomo et al., 2016). Cysteine proteases (CPs) and amoebapores (APs) have been shown to be essential for tissue invasion and abscess progression (Thibeaux et al., 2012). Among 50 CP genes that are encoded in the genome of *E. histolytica*, only three, CP-A1, CP-A2, and CP-A5, are highly expressed in steady state and account for 90% of the CP activity in trophozoite lysates (Irmer et al., 2009). APs are ~100 amino acid (aa) small peptides containing the “saposin-like protein” (SAPLIP) domain (Nowak et al., 2004). CPs and APs are stored in the lysosomes of trophozoites (Saito-Nakano et al., 2004) and are secreted in a constitutive and contact-dependent manner with host cells, respectively (Leippe et al., 1995). Anti-sense repression of CP-A5 (Ankri et al., 1999) or AP-A (Zhang et al., 2004) caused decreased abscess size, suggesting that both factors are essential for abscess formation.

Membrane traffic is mediated by mechanisms that are highly conserved among eukaryotes. It is controlled by two major families of small GTPases and their accessory molecules (Hutagalung and Novick, 2011). The Arf and Rab families of GTPases are involved in vesicular budding and fusion, respectively, and their functions are regulated according to the status of the bound guanine nucleotides (GTP-bound active or GDP-bound inactive state) (Hutagalung and Novick, 2011; Jackson and Bouvet, 2014). The attachment of vesicles to the target membrane is promoted by the membrane-targeted Rab-regulated tethering complex. Subsequently, membrane fusion finally occurs by the action of soluble *N*-ethylmaleimide-sensitive factor (NSF) attachment protein receptors (SNAREs) and their associated regulatory components (Ungermann and Langosch, 2005). The *Entamoeba* genome apparently contains more than 100 *Rab* genes. The number is comparable to or sometimes higher than those in multicellular eukaryotes (Saito-Nakano et al., 2005; Nakada-Tsukui et al., 2010). Due to their complexity and heterogeneity, only nine *E. histolytica* Rab proteins have been analyzed for their functions and shown to be involved in pathogenesis at least *in vitro*. EhRab5, EhRab7A, EhRab8A, EhRabA, and EhRabB were shown to be involved in phagocytosis and trogocytosis (Saito-Nakano et al., 2004; Nakada-Tsukui et al., 2005; Welter and Temesvari, 2009; Juarez-Hernandez et al., 2013; Hanadate et al., 2016). EhRab7A, EhRab7B, EhRab7D, and EhRab11B were shown to play a role in the intracellular CP transport and maturation (Nakada-Tsukui et al., 2005; Mitra et al., 2007; Saito-Nakano et al., 2007; Saito-Nakano et al., 2021). EhRab21 was demonstrated to be associated with attachment actin cytoskeleton reorganization (Emmanuel et al., 2015). EhRabB is the only Rab protein that was implicated in the virulence *in vivo* and *in vitro*. It was shown that expression of the dominant-negative mutant of EhRabB caused a reduction of phagocytosis *in vitro* and reduced the size of liver abscesses in the hamster model (Juarez-Hernandez et al., 2013). While biological roles of the representative Rabs have been elucidated, those of Arfs remain largely elusive.

In the present study, we describe the identification and characterization of one member of Arf GTPase family, EhArfX2. We identified *EhArfX2* as one of the highly upregulated genes in the hamster liver-passed virulent strain when compared to the isogenic laboratory-maintained attenuated strain. We further demonstrated that EhArfX2 and the EhArfX2-mediated traffic pathway are involved in the defense against nitrosative stress, which is known to occur during liver abscess formation. We have further demonstrated that EhArfX2 is localized to the trans-Golgi network and involved in trafficking from the trans-Golgi network to lysosomes. Finally, we demonstrated the *in vivo* role of EhArfA2; expression of dominant-negative mutant EhArfX2 caused a decrease in the formation of liver abscesses in hamsters, which was likely attributable to decreased resistance to nitrosative stress.

## Materials and Methods

### Ethics Statement

The animal procedures were approved by the Institutional Animal Care and Use Committee (No. 211075) and conducted at the Association for Assessment and Accreditation of Laboratory Animal Care (AAALAC)-accredited National Institute of Infectious Diseases, Japan.

### Cell Culture

Trophozoites of the *E. histolytica* strain-attenuated HM-1:IMSS Cl6 (a-HM1) were cultured axenically at 35°C in BI-S-33 medium, as previously described (Diamond et al., 1972; Diamond et al., 1978). Liver-passed virulent HM-1 (v-HM1) was cultured in YIMDHA-S medium supplemented with live *Crithidia fasciculata* at 35°C (Kobayashi et al., 2005).

### Transcriptome Analysis

The Affymetrix (Santa Clara, CA, USA) custom array containing probe sets for 9,435 open reading frames (ORFs) from the *E. histolytica* genome database was used (Gilchrist et al., 2006). Synthesis of cDNA, hybridization, washing, staining, scanning of the arrays, and data acquisition (Gene Chip scanner 3000I, Affymetrix) were performed according to standard Affymetrix protocols (Gilchrist et al., 2006). Differentially expressed genes were identified by the following criteria: a fold change of at least 2-fold upregulated or downregulated in the virulent strain (v-HM1) as compared to the attenuated strain (a-HM1), a raw signal intensity of >10, a standard deviation of triplicates of the normalized signal intensity of <0.5, and a p-value of Student’s t-test of <0.05.

### Quantitative Real-Time PCR

The mRNA expression of *EhArf* and *EhSar* genes was analyzed by quantitative real-time PCR analyses, essentially as previously described (Gilchrist et al., 2006; Saito-Nakano et al., 2007), with some modifications (Applied Biosystems 7500, Foster City, CA, USA). RNA polymerase II served as an internal control (GenBank accession number XP_649091). The primers used are listed in **Supplementary Table S1**. The following parameters were used: an initial denaturation step at 95°C for 9 min, followed by 40 cycles of denaturation at 94°C for 30 s, annealing at 50°C for 30 s, and extension at 65°C for 1 min. A final step at 95°C for 9 s, 60°C for 9 s, and 95°C for 9 s was used to remove primer dimers.

### Collection of EhArf GTPases and EhSNARE Orthologs

The *E. histolytica* genome database was searched using *S. cerevisiae* and *Homo sapiens* Sar, Arf, and Arl proteins as queries by BLASTP. The *E. histolytica* genome database was also searched by BLASTP using EhSar1a, EhArfX1, and EhArfX2. All the possible *Entamoeba* Sar/Arf/Arl protein sequences were reexamined with BLASTP analysis using individual amoebic protein as an inquiry sequence against the human database at the National Center of Biotechnology Information (NCBI) as reported previously (Saito-Nakano et al., 2005; Nakada-Tsukui et al., 2010).

### Phylogenetic Analysis

Multiple alignment of sequences of EhArf GTPases was obtained using MUSCLE (Edgar, 2004) and was further corrected by manual inspection. With 22 sequences, 152 unambiguously aligned aa sites were used for the below analysis, corresponding to residues 5–10, 14–92, 94–113, 115–130, and 145–175 of the EhArfX2 sequence. Phylogenetic analyses by the maximum likelihood (ML), neighbor-joining (NJ), and maximum parsimony (MP) methods were performed using PROML, PROTDIST + NEIGHBOR, and PROTPARS programs, respectively, in the PHYLIP package (http://evolution.gs.washington.edu/phylip.html). To analyze the ML and NJ methods with the JTT model considering heterogeneous substitution rates across sites (the JTT + G model), the Γ-shape parameter of the discrete Γ-distribution with four categories that approximated site rates was estimated using the CODEML program in PAML package (Yang, 1997) and the ML best tree with homogeneous substitution rates across sites. Bootstrap analysis for each of the three methods was performed by the ML (the JTT + G model), NJ (the JTT + G model), or MP methods to the resampled data sets produced by the SEQBOOT programs in the PHYLIP package. One thousand resamplings were performed for all analyses. A consensus tree was generated using the CONSENSE program, in the PHYLIP package, based on the bootstrap analysis of the ML method. The AU test in the CONSEL program was used for statistical comparisons among the alternative trees (Shimodaira, 2002). The alternative trees for the test were prepared by grafting EhArfX2 branch to all branches of the backbone tree.

### Plasmid Construction

To construct plasmids expressing C-terminus 3HA-tagged or 3myc-tagged EhArfX2, the *EhArfX2* coding region was PCR amplified from cDNA synthesized from a-HM1 and inserted in the BglII site of pEhExHA or pKT-3M vector, respectively (Saito-Nakano et al., 2004; Nakada-Tsukui et al., 2005). Plasmids expressing EhArfX2^Q68L^ and EhArfX2^T28N^ mutants were constructed by PCR-mediated mutagenesis using a PrimeSTAR Mutagenesis Basal Kit (Takara, Japan). To construct plasmids expressing green fluorescence protein (GFP)-fusion protein, fragments coding EhGalT, EhSed5, and EhYkt6 were amplified from cDNA and inserted in the pEhExGFP vector (Watanabe et al., 2020), which was removed myc-tagged sequence from a previously reported myc-GFP fusion-expressing vector, pKT-MG (Saito-Nakano et al., 2004). The necessary information for plasmid construction is shown in **Supplementary Table S1**. To generate a vector for double-expressing CP-A5-HA and 3myc-tagged EhArfX2, a 1.7-kb fragment containing the 3myc-EhArfX2 coding region-flanked 5’ upstream and 3’ downstream untranslated regions (UTRs) was cloned into the SpeI site of CP-A5-HA-expressing plasmid (Sato et al., 2006a) as described previously (Saito-Nakano et al., 2004). Oligonucleotide sequences used for the plasmid construction were listed in **Supplementary Table S1**.

### Amoeba Transformants

Plasmids were introduced into the a-HM1 and v-HM1 strains by lipofection as previously described (Nozaki et al., 1999). Transformant amoebae were selected by the addition of 1 µg/ml Geneticin after 24-h transfection and gradually increased to 6 µg/ml for 2 weeks.

### Antibodies

Anti-Bip (Hanadate et al., 2016), anti-Vps26 (Nakada-Tsukui et al., 2005), anti-CPBF1 (Furukawa et al., 2012), and anti-CS1 (Okada et al., 2006) antibodies were previously described, respectively. Anti-GFP, anti-HA, and anti-Myc antibodies were purchased as follows: rabbit anti-GFP, A11122 (Molecular Probes, Invitrogen, Carlsbad, CA, USA); mouse anti-GFP, 11814460001 (Roche Diagnostics, Indianapolis, IN, USA); mouse anti-HA, clone 16B12, and rabbit anti-Myc, clone A-14 (Santa Cruz Biotech, Santa Cruz, CA, USA).

### Indirect Immunofluorescence Analysis

An indirect immunofluorescence assay was conducted essentially as previously described (Saito-Nakano et al., 2004). Trophozoites were attached to 8-mm round wells on a glass slide, fixed with 3.7% paraformaldehyde, permeabilized with 0.1% Tx100, and reacted with antibodies. Alexa 488- and Alexa 568-conjugated immunoglobulin G (IgG; Molecular Probes) were used as secondary antibodies. Acidic compartments of trophozoites were stained with 500-fold dilution of LysoTracker Red DND-99 (Invitrogen) for 12 h. Images were acquired using LSM510 or LSM780 confocal laser scanning microscope (Zeiss, Germany). Colocalization analyses were processed using Fiji software (Schindelin et al., 2012).

### Subcellular Fractionation

The subcellular fractionation was performed as reported previously with some modifications (Saito-Nakano et al., 2007; Hanadate et al., 2016). Approximately 3 × 10^5^ amoeba cells were washed with cold phosphate buffered saline (PBS) containing 2% glucose, resuspended in homogenization buffer (250 mM sucrose, 50 mM Tris, pH 7.5, 50 mM NaCl, 0.1 mg/ml of E-64), and homogenized on ice with 30 strokes with a Dounce homogenizer. After unbroken cells were removed by centrifugation at 400 × *g* for 2 min, the supernatant was centrifuged at 13,000 × *g* at 4°C for 10 min to obtain the pellet (p13) and supernatant (s13) fractions. The s13 fraction was further separated by centrifugation at 100,000 × *g* at 4°C for 1 h to obtain soluble (s100) fractions. These fractions were subjected to immunoblot analyses with anti-HA, anti-CPBF1, or anti-CS1 antibodies.

### Cysteine Protease Assay

CP activity in the culture supernatant and parasite lysates was assayed as previously described (Saito-Nakano et al., 2007). CP activity was measured using z-Arg-Arg-7-amino-4-trifluoromethylcoumarin substrate as described (Leippe et al., 1995).

### Phagocytosis Assay

Phagocytosis of erythrocytes was described previously (Saito-Nakano et al., 2004). Amoeba trophozoites were attached to 8-mm round wells on a glass slide and then incubated with 1 × 10^7^ erythrocytes/ml in biosate, yeast extract, iron-serum (BIS) medium under the indicated incubation times at 37°C. Cells were fixed with paraformaldehyde, ingested erythrocytes were stained with diaminobenzidine, and EhArfX2-HA mutant-expressing trophozoites were stained with anti-HA antibody. The number of ingested erythrocytes in EhArfX2-HA-expressing trophozoites was counted under the microscope.

### Hamster Liver Abscess Model

Approximately 4 × 10^5^ v-HM1 were injected into the left lobe of the liver of 5-week-old Syrian hamsters. Animals were sacrificed 6 days post infection, and the liver and abscess were dissected and weighed separately (Sato et al., 2006b).

### Assays for Sensitivity to Nitric Oxide and H_2_O_2_

EhArfX2 transformants established in the v-HM1 strain were seeded in 96-well plates at 1 × 10^4^ cells/well in the presence of the indicated concentration of drugs with BIS medium. After 18-h culture, the medium was removed and the viability of attached trophozoites was measured by the addition of 10% WST-1 reagent (Roche) in Opti-MEM medium for 20 min at 37°C (Penuliar et al., 2015). The absorbance at 450 nm was measured using a DTX880 Multimode Detector (Beckman Coulter, Brea, CA, USA). Experiments were repeated three times with triplicate replicates per experiment.

## Results

### Comparative Transcriptomic Analysis of Animal-Passaged Virulent and Culture-Attenuated HM-1 Strains Led to Identification of Upregulated and Downregulated *E. histolytica* Genes

To identity genes that are involved in virulence *in vivo*, we exploited comparative transcriptome analysis using a custom-made microarray containing probe sets for all ORFs from the *Entamoeba* genome database, which we previously designed and used in a number of studies (Gilchrist et al., 2006; Husain et al., 2011; Penuliar et al., 2012; Penuliar et al., 2015; Jeelani et al., 2017). Gene expression profiles were compared between two isogenic strains with the identical genetic background but maintained in different ways: one that had been passaged through the hamster liver (virulent HM-1:IMSS Cl6 strain, v-HM1 hereinafter) and one that had been continuously cultured *in vitro* and was no longer capable of producing abscesses in hamsters (a-HM1) (see **Figure 4C**, described below). The transcriptomic comparison of v-HM1 and a-HM1 identified 59 genes that were differentially expressed (>4-fold, *q*-value <0.05) with the significance of gene expression was determined using the significance analysis of microarray statistical program (https://tibshirani.su.domains/SAM/) (Gilchrist et al., 2006). Among them, 30 genes were upregulated (**Table 1**) and 29 were downregulated in v-HM1 (**Supplementary Table S2**). Among the 30 upregulated genes, three genes appear to be associated with cellular functions related to membrane traffic based on annotation: two Arf GTPases (EHI_189960, EHI_073480) and ATG4 cysteine peptidase (EHI_055660). Neither Rab GTPase nor SNARE was identified as upregulated or downregulated genes. In this study, we describe our analysis of the Arf GTPases that we identified by the comparative transcriptomic analysis. Characterization of other upregulated and downregulated genes will be reported elsewhere.

**Table 1:** List of genes upregulated in v-HM1.

Probe Set	GenBank Acc. #	EHI No.	Fold Change (Log2)	Gene Annotation
**Intracellular trafficking**				
122.m00139	XP_652330	EHI_189960	5.4	Arf GTPase (EhArfX2)
9.m00410	XP_656676	EHI_073480	2.7	Arf GTPase (EhArfX3)
134.m00147	XP_652043	EHI_055660	2.2	ATG4 cysteine peptidase
**Signal transduction**				
2.m00588	XP_657408	EHI_151730	3.6	Protein kinase
484.m00039	XP_648310	EHI_023860	2.7	Protein kinase
64.m00165	XP_654053	EHI_137810	2.6	Tyrosine kinase
94.m00141	XP_653083	EHI_086050	2.3	Tyrosine kinase
**Metabolism**				
101.m00114	XP_652901	EHI_059910	3.8	Acid phosphatase
128.m00121	XP_652205	EHI_109890	3.8	Chitinase
11.m00315	XP_656535	EHI_042260	2.1	NADP-dependent alcohol dehydrogenase
**Transcription and translation**				
81.m00165	XP_653489	EHI_022950	2.3	DnaJ family protein
129.m00147	XP_652177	EHI_127170	2.1	Elongation factor-2 kinase
20.m00296	XP_655916	EHI_177460	3.9	Translation initiation factor eIF-5A
**Hypothetical**				
31.m00224	XP_655335	EHI_012440	10.3	Hypothetical protein
9.m00413	XP_656679	EHI_073450	7.3	Hypothetical protein
42.m00175	XP_654870	EHI_124550	4.1	Hypothetical protein
12.m00306	XP_656478	EHI_187080	3.2	Hypothetical protein
25.m00235	XP_655627	EHI_023440	2.9	Hypothetical protein
6.m00458	XP_656937	EHI_096610	2.9	Hypothetical protein
298.m00065	XP_649480	EHI_113930	2.7	Hypothetical protein
45.m00159	XP_654749	EHI_199710	2.7	Hypothetical protein
2.m00587	XP_657407	EHI_151740	2.7	Hypothetical protein
124.m00116	XP_652284	EHI_024550	2.7	Hypothetical protein
33.m00241	XP_655226	EHI_136480	2.6	Hypothetical protein
22.m00288	XP_655823	EHI_150420	2.5	Hypothetical protein
163.m00092	XP_651472	EHI_119510	2.3	Hypothetical protein
128.m00122	XP_652206	EHI_109880	2.2	Hypothetical protein
101.m00117	XP_652893	EHI_059900	2.2	Hypothetical protein
16.m00352	XP_656170	EHI_185250	2.0	Hypothetical protein
20.m00282	XP_655901	EHI_137990	2.0	Hypothetical protein

### Verification of Specific Overexpression of EhArfX2 in a Virulent Strain of *E. histolytica*

**Figure 1:**
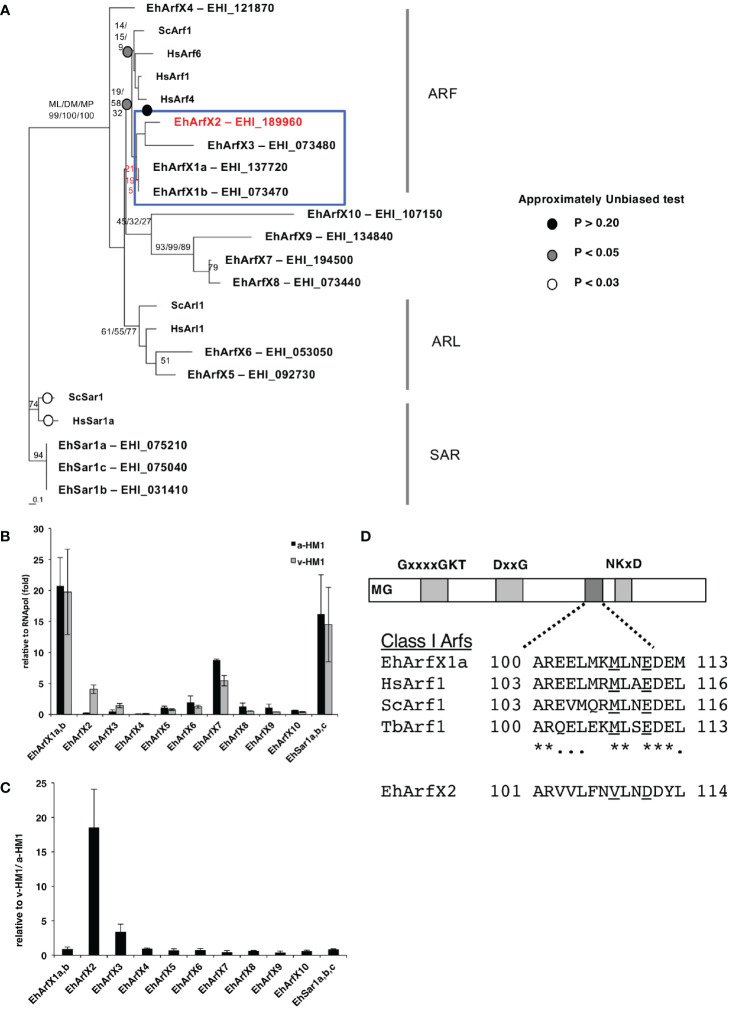
Phylogenetic position, mRNA expression, and structure of *E*. *histolytica* Arf proteins. **(A)** The consensus maximum likelihood (ML) tree of 14 members of the Sar/Arf family of GTPases from *E*. *histolytica* and representative members from yeast and human. Bootstrap probabilities by the ML method are attached to the internal branches. Branches with less than 50% bootstrap support are unmarked. For the node of interest, bootstrap values determined by the distance matrix (DM) and maximum parsimony (MP) methods are also shown. Circles highlighted by white, gray, and black indicate the branches in which *p*-value by the AU test is more than 0.05. The branches in which *p*-value is less than 0.01 are unmarked. **(B)** Relative abundance of mRNA of 14 *E*. *histolytica* Sar/Arf genes. Quantitative real-time PCR was performed using cDNA synthesized with total RNA from a-HM1 and v-HM1 as templates. As EhArfX1a (EHI_137720) and EhArfX1b (EHI_073470) shared 99.8% nucleotide identity, and three EhSar1 genes (EHI_075210, EHI_075040, EHI_031410) shared 99.8% nucleotide identities, it was impossible to distinguish each isotype. Bars indicate standard deviation (n = 3). **(C)** Normalized mRNA expression levels of 14 *E*. *histolytica* Sar/Arf genes in v-HM1 relative to a-HM1. Bars indicate standard deviation (n = 3). **(D)** Schematic diagram of class I Arf proteins. EhArfX2 lacks the Golgi-targeting motif that is observed in EhArfX1 and Arf1 from other organisms. The consensus MxxE motif is underlined. *conserved amino acids.

The *E. histolytica* genome contains genes encoding 14 members of the Sar/Arf family of GTPases based on the *E. histolytica* genome database at AmoebaDB (http://amoebadb.org/amoeba/, version 54 released on September 8, 2021). Phylogenetic analysis revealed none of the *E. histolytica* Sar/Arf family GTPases has close kinship with known human and yeast counterparts at statistically supported levels (bootstrap values >70 by at least one of the following analyses: ML, DM, and MP) (**Figure 1A**). Thus, assignment of orthologs is not possible. We alternatively assigned *E. histolytica Arf* genes with “X” as a prefix, as all genes appear to be lineage specific. *E. histolytica* Arfs were numerically designated in a descending order of the percentage identity to human Arf1 with the “X” prefix (**Supplementary Table S3**). In a case where two *E. histolytica* Arf proteins show >99% identity, they are considered to be allelic isotypes and designated with alphabets in lowercase, e.g., EhArfX1a and EhArfX1b. EhArfX5 and EhArfX6 form monophyly and are positioned closely to human and yeast Arl1, although not statistically supported. EhArfX7–EhArfX9 also form monophyly and represent an *E. histolytica-*specific clade (**Figure 1A**). Three allelic isotypes of EhSar1a–EhSar1c form a well-supported monophylic clade with >99% identity and show high similarity with human and yeast Sar1 (**Supplementary Table S3**). EhArfX1a/b, X2, and X3 appear to form monophyly, but the tree topology was not statistically supported.

The mRNA levels of *EhArfX2* and *EhArfX3* in a-HM-1 were relatively low when compared to other members of 14 *Sar/Arf* GTPase family genes, as suggested by our microarray analysis and further validated by quantitative real-time PCR (**Figure 1B**). In v-HM1, mRNA levels were higher compared to a-HM-1: 18 ± 5.5-fold for *EhArfX2* and 3.4 ± 1.1-fold for *EhArfX3*, respectively (**Figure 1C**). In the subsequent study, we focused on the biological and pathogenic roles of EhArfX2.

### Structural Features of *E. histolytica* EhArfX2

Among the Arf subfamily of GTPases, the function of Arf1, which is primarily localized to the Golgi apparatus in humans, yeasts, *Trypanosoma brucei*, and *Arabidopsis thaliana* (Teal et al., 1994; Price et al., 2007; Matheson et al., 2008), has been well studied. It was shown that the Golgi targeting of Arf1 is regulated *via* interaction of the MxxE motif present in the second (DxxG) and third (NKxD) GTP-binding consensus sequences (Honda et al., 2005) (**Figure 1D**). Although EhArfX1a does not show high similarity to Arf1 based on protein alignment and phylogenetic analysis (**Figure 1A**), the MxxE motif is well conserved in EhArfX1a and EhArfX1b (**Figure 1D**). EhArfX1a and EhArfX1b may be the functional orthologs of mammalian Arf1. In contrast, EhArfX2 does not possess the MxxE motif, and subcellular localization and function of EhArfX2 could not be presumed *in silico*.

### EhArfX2 Is Colocalized With the *E. histolytica* Ortholog of Trans-Golgi SNARE EhYkt6 but Not With Either Cis-Golgi or Endoplasmic Reticulum Markers

**Figure 2:**
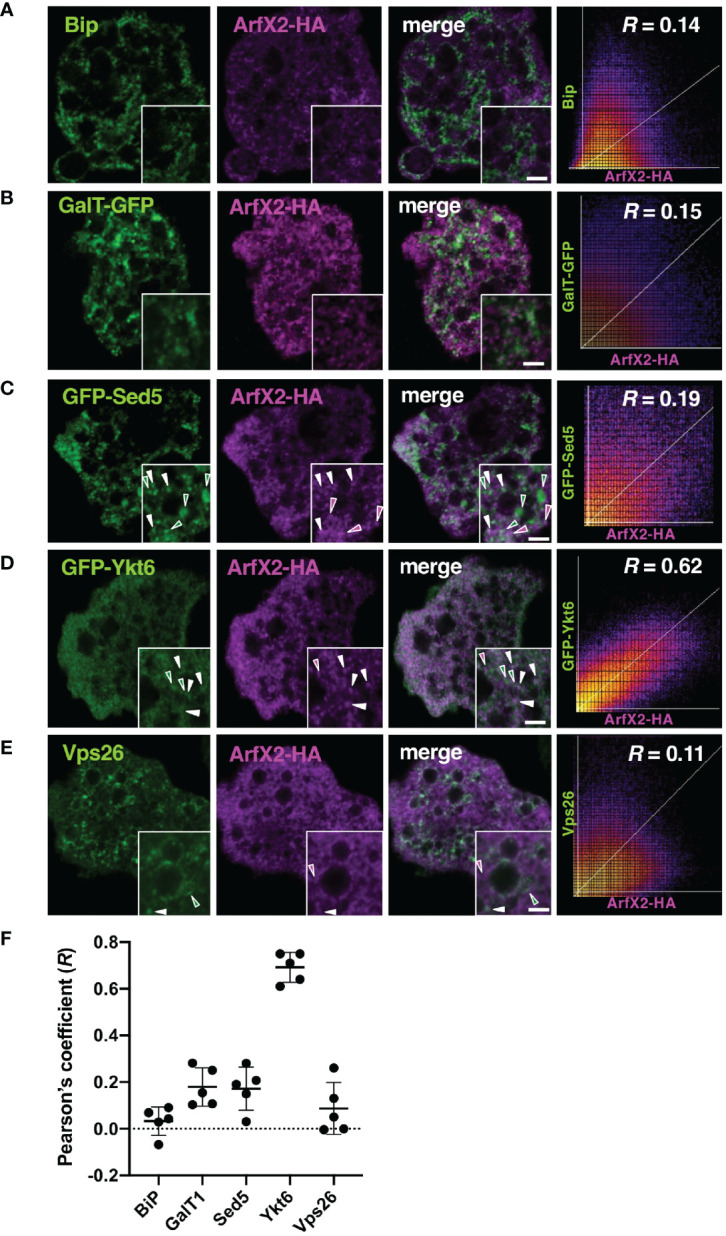
Subcellular localization of EhArfX2-HA and other organelle markers. EhArfX2-HA-expressing cells were stained with anti-HA antibodies (magenta) and anti-Bip **(A)** or anti-Vps26 antibodies **(E)**, respectively (green). EhArfX2-HA and EhGalT-GFP double-expressing **(B)**, EhArfX2-HA and GFP-EhSed5 double-expressing **(C)**, or EhArfX2-HA and GFP-EhYkt6 double-expressing **(D)** cells were stained with anti-GFP and anti-HA antibodies. Colocalized vesicular signals are indicated with white arrowheads. Scatter plots depict colocalization analysis. *R* = Pearson’s correlation coefficient. Thick bars, 2 µm. **(F)** The R values obtained from panels **(A–E)** are plotted, and average (bold bars) and standard deviation (thin bars) are indicated (n = 5).

The Arf family of GTPases is known to be localized to the Golgi apparatus, the trans-Golgi network, endosomes, and the plasma membrane in other organisms (Jackson and Bouvet, 2014). We examined the subcellular localization of EhArfX2 using ER and the Golgi markers (Makiuchi and Nozaki, 2014). Indirect immunofluorescence assay using the transformant expressing GFP fused with the signal peptide (SP) from Gal/GalNAc lectin at the N-terminus and ER retention signal (KDEL) at the C-terminus (SP-GFP-KDEL) (Teixeira and Huston, 2008) with antiserum against *E. histolytica* Bip, the ER luminal chaperon (EHI_199890) (Munro and Pelham, 1986; Hanadate et al., 2016), revealed colocalization with high Pearson’s correlation coefficient (R = 0.67; **Supplementary Figure S1A**). While indirect immunofluorescence assay using the transformant expressing EhArfX2 fused with the HA tag at the C-terminus (EhArfX2-HA), anti-HA and anti-Bip antibodies, revealed different staining patterns (R = 0.14; **Figure 2A**). Next, an *E. histolytica* ortholog of Golgi-luminal galactosyl transferase (EhGalT), which has the N-terminal SP but lacks a transmembrane domain, was used for the Golgi luminal marker [EHI_000660, 45% aa identity to *Strongylocentrotus purpuratus* (sea urchin) galactosyl transferase, e-value = 3e-6]. GalT presumably, by analogy, catalyzes a transfer of galactose, N-acetylgalactosamine, and N-acetylglucosamine in the Golgi lumen (Magnelli et al., 2008). HA-tagged EhGalT, detected with anti-HA antibody, showed dot-like structures that were well associated with ER stained by anti-Bip antibody (R = 0.74; **Supplementary Figure S1B**). The high-resolution 3D image revealed that the dot-like EhGalT signals were associated with the continuous Bip signal (**Supplementary Figure S1C**). The distribution of EhArfX2-HA detected with anti-HA antibody differed from the pattern of EhGalT-GFP detected with anti-GFP antibody (R = 0.15; **Figure 2B**).

We further attempted to localize EhArfX2 by examining if EhArfX2 is colocalized with some organelle-specific SNAREs, which are known to mediate membrane fusion together with Rab GTPases (Hong, 2005). In membrane fusion, the coiled-coil SNARE motif of R-SNAREs on the acceptor vesicle membrane (previously called v-SNARE) makes specific interaction with the SNARE motif of Q-SNARE (or called t-SNARE) on the donor membrane to form a cognate complex. The SNARE motif is ubiquitous and evolutionally conserved throughout eukaryotic lineages (Hong, 2005; Kloepper et al., 2007). We identified 25 SNARE, 16 Q-SNARE, and nine R-SNARE genes in the *E. histolytica* genome (http://amoebadb.org/amoeba/, version 54 released on September 8, 2021) by the genome wide survey using the conserved coiled-coil SNARE motif domain, followed by phylogenetic analysis (**Supplementary Figures S2A**, **S3A**). The description of the entire repertoire of SNAREs will be reported elsewhere. Among them, two SNAREs were used in this study as cis-Golgi and trans-Golgi markers. EHI_181290 is an *E. histolytica* ortholog of cis-Golgi SNARE Sed5 from *S. cerevisiae* and *A. thaliana* (also annotated as syntaxin-5 in human). The mutual overall aa identity of the Sed5 orthologs among organisms such as yeasts, plants, and mammals is low (31%–37%, e-value = 8e-33–2e-48); similarly, it is also low between *E. histolytica* Sed5 ortholog, EhSed5 (EHI_181290), and orthologs from these organisms (e.g., 28% aa identity to human syntaxin-5, e-value = 3e-18) (**Supplementary Figure S2A**). Despite the low level of aa conservation, all known Sed5 orthologs are known to localize to the cis-Golgi membrane, warranting validation as the cis-Golgi marker (Shestakova et al., 2007; Chatre et al., 2009). Three Golgi-associated signatures that were identified in Sed5 from other organisms are well conserved in EhSed5: the short stretch of aa close to the N terminus (RDRTxEF) (Yamaguchi et al., 2002), the coiled-coil SNARE domain of ~60 aa close to the C terminus, and the short (15 aa) C-terminal transmembrane segment (**Supplementary Figure S2B**). It was established that the RDRTxEF motif is required for the SNARE complex assembly and functions in ER–Golgi fusion (Yamaguchi et al., 2002). Compared to the plasma membrane-localized syntaxins, which typically contain a relatively long (the average of 23 aa) transmembrane segment, the transmembrane region of Sed5 and Syn5 from yeast and human, respectively, is shorter and rich in phenylalanine (**Supplementary Table S4**) (Bretscher and Munro, 1993; Banfield et al., 1994). On the other hand, EHI_052110 is an apparent *E. histolytica* ortholog of trans-Golgi SNARE Ykt6 (37% aa identity to human Ykt6, e-value = 3e-23) (Tai et al., 2004; Chen et al., 2005) and used as a trans-Golgi marker (**Supplementary Figure S3A**). Unlike other SNARE members, Ykt6 lacks a transmembrane domain but possesses a conserved C-terminal dicysteine motif, which is modified by farnesylation and geranylgeranylation (Sakata et al., 2021) (**Supplementary Figure S3B**). EhYkt6 showed good colocalization with EhArfX2-HA (R = 0.62) (**Supplementary Figure S4A**), whereas EhSed5 did not (R = 0.19) (**Figures 2C, D**). The subcellular localization of EhSed5, Bip, and EhYkt6 was clearly distinct (R < 0.1; **Supplementary Figures S4B, C**). The localization of EhArfX2 was also different from the EhVps26-positive endosomal-like compartment (Nakada-Tsukui et al., 2005) (**Figure 2E**). These results indicate that EhArfX2 showed the highest colocalization with EhYkt6 among ER and the Golgi markers (**Figure 2F**) and localizes to the trans-Golgi or its equivalent compartment in *E. histolytica*.

### EhArfX2 Is Involved in Lysosomal Trafficking and Biogenesis

**Figure 3:**
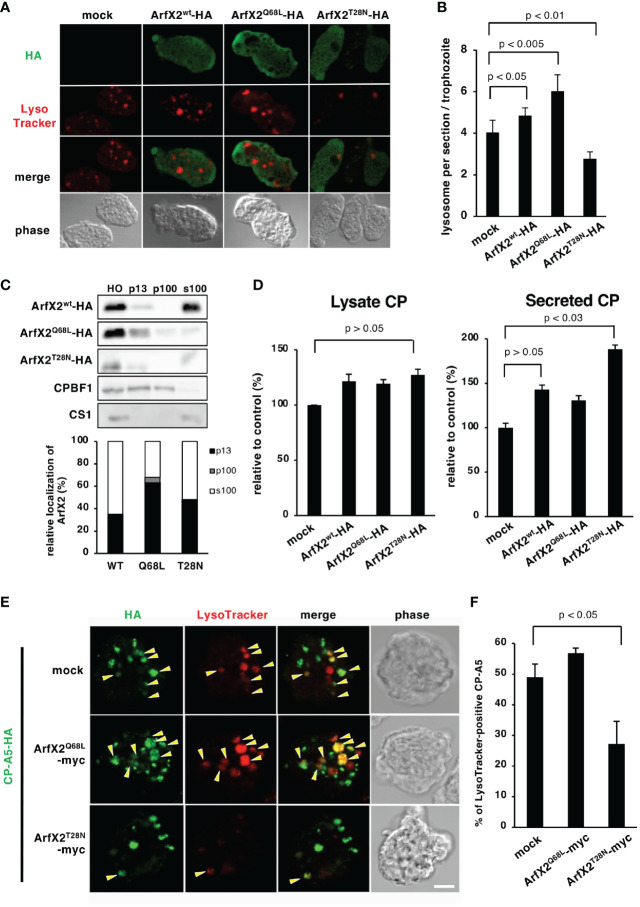
Effects of expression of EhArfX2 wild type and mutants on lysosome trafficking. **(A)** Indirect immunofluorescence assay of EhArfX2^WT^-HA, EhArfX2^Q68L^-HA, and EhArfX2^T28N^-HA. EhArfX2 and lysosomes were stained with anti-HA (green) and LysoTracker Red (red), respectively. **(B)** Number of lysosomes in EhArfX2^WT^-HA-, EhArfX2^Q68L^-HA-, and EhArfX2^T28N^-HA-expressing transformant cells. Lysosomes were visualized with LysoTracker Red, and the number of lysosomes per confocal section was counted in independent 30 trophozoites. **(C)** Subcellular fractionation of EhArfX2^WT^-HA, EhArfX2^Q68L^-HA, and EhArfX2^T28N^-HA. Homogenate (HO) was separated into low-speed pellet (13,000 × g, p13), high-speed pellet (100,000 × g, p100), and soluble (100,000 × g, s100) fractions, respectively (upper panel). EhArfX2-HA was probed with anti-HA antibody. Antibodies against CP-binding protein family 1 (CPBF1) and cysteine synthase 1 (CS1) were used as a marker of membrane and cytosol fractions, respectively. Quantification of EhArfX2-HA in the p13, p100, and s100 fractions by chemiluminescence measurement (lower graph). **(D)** CP activity in the culture medium (left) and cell lysates (right) in the control (mock), EhArfX2^wt^-HA-, EhArfX2^Q68L^-HA-, and EhArfX2^T28N^-HA-expressing cells. **(E)** Localization of HA-tagged CP-A5 in EhArfX2^Q68L^-myc- and EhArfX2^T28N^-myc-expressing cells. CP-A5-HA and lysosomes were stained with anti-HA antibody (green) and LysoTracker Red (red), respectively. LysoTracker-positive CP-A5-HA signals are indicated by yellow arrowheads. Scale bar, 10 µm. **(F)** Relative colocalization of CP-A5-HA and LysoTracker. The number of LysoTracker and CP-A5-HA double-positive compartments and the total number of CP-A5-HA-positive compartments in the cells were counted (n = 30 cells). The percentages of the double-positive compartments per total number of CP-A5-HA-positive compartments are shown. Significance was evaluated by the Student’s t-test.

We further investigated the trafficking pathway mediated by EhArfX2, the amoeba transformants that expressed either a constitutively active GTP-bound or an inactive GDP-bound form of EhArfX2 mutant, which corresponds to human Arf1^Q71L^ or Arf1^T31N^, respectively (Dascher and Balch, 1994; Zhang et al., 1994). We took advantage of an approach of dominant-negative mutant because gene repression by small antisense RNA-mediated transcriptional gene silencing (Morf et al., 2013; Nakada-Tsukui et al., 2018) was not feasible based on the fact that *EhArfX2* gene was not expressed in a-HM1, and neither in G3 strain, which is commonly used for gene silencing (**Figure 1B**). We assumed that EhArfX2 may be involved in trafficking from the trans-Golgi to lysosomes. The dominant-active EhArfX2^Q68L^ and dominant-negative EhArfX2^T28N^ mutants fused with the HA-tag on the C-terminus were constitutively expressed (**Figure 3A**). This episomal plasmid-driven expression caused up to 10–58-fold increase in the sum (i.e., intrinsic plus exogenous) of the steady-state *EhArfX2* mRNA in a-HM-1 (**Supplementary Figure S5**), and the sum level of the *EhArfX2* transcripts was comparable to the level of *EhArfX2* mRNA in v-HM1 (**Figure 1C**). Indirect immunofluorescence assay using anti-HA antibody showed that both HA-tagged EhArfX2^Q68L^ and EhArfX2^T28N^ mutant proteins were distributed to small vesicles and the cytosol, similar to that of wild-type EhArfX2-HA (**Figure 3A**). The expression of EhArfX2 mutants affected the number of lysosomes visualized with LysoTracker Red. The number of lysosomes per section was increased in EhArfX2^wt^ (4.8 ± 0.4 lysosomes per section, *p* < 0.05 compared to mock control) and EhArfX2^Q68L^ (6.0 ± 0.7, *p* < 0.005) compared to mock control (4.0 ± 0.5). On the contrary, the number of lysosomes was decreased by the expression of EhArfX2^T28N^ (2.7 ± 0.3 lysosomes per section, *p* < 0.01) (**Figure 3B**). Subcellular fractionation of cell lysates followed by immunoblot analysis showed that the amount of EhArfX2^WT^-HA in the cell lysate and the soluble fraction (s100) were much higher than those of EhArfX2^Q68L^ and EhArfX2^T28N^-HA (**Figure 3C**). A fraction of EhArfX2^WT^-HA, EhArfX2^Q68L^-HA, and EhArfX2^T28N^-HA was also distributed to the membrane-associated fractions (p13 and p100). A majority of EhArfX2^Q68L^-HA protein (68% ± 4%) was associated with the membrane (p13 and p100 fractions), while 35% ± 6.7% EhArfX2^wt^-HA and, unexpectedly, 48% ± 16% EhArfX2^T28N^-HA were partitioned to the membrane-associated fractions (**Figure 3C**). These data are consistent with a premise that EhArfX2 cycles between small vesicular membranes and the cytosol in a nucleotide-dependent manner and is involved in lysosome biogenesis.

### EhArfX2 Is Involved in Cysteine Protease Transport and Maturation

CPs were shown to be involved in a variety of biological processes in *E. histolytica* including degradation of mucin and extracellular matrix (Moncada et al., 2006), target cell cytolysis (Thibeaux et al., 2012), inactivation of immune anaphylatoxins, immune evasion, and implicated in tissue invasion in the colon and the liver (Reed et al., 1995; Ankri et al., 1999; Moncada et al., 2006; Thibeaux et al., 2012; St-Pierre et al., 2017). It was shown that CPs are stored in lysosomes and constitutively secreted to the culture medium (Leippe et al., 1995; Saito-Nakano et al., 2004; Sato et al., 2006a). We examined whether overexpression of EhArfX2^WT^ or mutants affects the lysosome biogenesis and lysosomal functions, more specifically, intracellular storage and secretion of CPs. EhArfX2^T28N^-expressing cells, which showed a defect in lysosome formation, showed 1.8 ± 0.2-fold increased secretion of CP to the medium when compared to the mock transformant cells (**Figure 3D**, right). The secretion of CP also appeared to be increased, although statistically not proven to be significant, in EhArfX2^WT^- and EhArfX2^Q68L^-expressing transformants (**Figure 3D**, right). On the contrary, the total amount of CP activity in lysates was not changed (**Figure 3D**, left). Increased secretion of CP in EhArfX2^T28N^ cells was not caused by the increase in the transcription levels of CP-A2 and CP-A5 mRNA because the transcript levels of CP-A2 and CP-A5 mRNA were comparable among the strains expressing EhArfX2 ^WT^ or mutants and mock strain (**Supplementary Figure S5**).

Interference of CP trafficking to lysosomes and subsequent missecretion of CPs by EhArfX2^T28N^ expression was further examined by the visualization of intracellular CPs. One of the major CPs implicated in pathogenesis, CP-A5, was tagged with HA at the C-terminus (Sato et al., 2006a) and co-expressed with either EhArfX2^Q68L^ or EhArfX2^T28N^ tagged with myc (**Figure 3E**). EhArfX2 with the C-terminal myc tag showed indistinguishable distribution from HA-tagged EhArfX2; EhArfX2-HA was also localized to small vesicular and cytosolic patterns (**Supplementary Figure S6**). CP-A5-HA was predominantly localized to lysosomes, stained with LysoTracker Red, in EhArfX2^Q68L^-expressing cells, as previously shown in the parental line (Sato et al., 2006a) (**Figure 3E**, mock). In EhArfX2^T28N^-expressing cells, 27% ± 7.3% of CP-A5-HA-positive compartments were stained with LysoTracker Red (**Figures 3E, F**). In contrast, in mock and EhArfX2^Q68L^ cells, 48% ± 4% and 56% ± 1% of CP-A5-positive compartments were LysoTracker Red positive, respectively (**Figure 3F**). These data indicate that expression of EhArfX2^T28N^ inhibits the intracellular transport of CP-A5 to lysosomes. In order to directly show that the traffic of lysosomal enzymes is regulated by EhArfX2, we monitored traffic and activity of CPs, which are the representative lysosomal enzymes and also the virulence-associated factor of the parasite. Gelatin substrate gel assay showed that the secretion of CPs to the medium increased in EhArfX2^WT^-HA, EhArfX2^Q68L^-HA, and EhArfX2^T28N^-HA strains (**Supplementary Figure S7A**). The intensity of the band corresponding to EhCP-A1 and the top band of two bands corresponding to EhCP-A2 appear to be increased in EhArfX2^WT^-HA, EhArfX2^Q68L^-HA, and EhArfX2^T28N^-HA strains. Note that EhCP-A2 was previously shown to localize to lysosomes (Saito-Nakano et al., 2007). Furthermore, destruction of the Chinese hamster ovary (CHO) monolayer by EhArfX2^T28N^-HA strain also increased compared to mock control strain (**Supplementary Figure S7B**). Furthermore, the increased cytopathic activity was canceled by CP inhibitor E64, validating that the observed cytopathic effect was attributable to CP activity. The gelatin substrate gel electrophoresis (Tillack et al., 2006) using culture supernatant of EhArfX2^T28N^-HA revealed increased secretion of EhCP-A2 (**Supplementary Figure S7A**), which is localized to the amoebic lysosomes (Saito-Nakano et al., 2007). Increased secretion of CP in EhArfX2^T28N^-HA was verified by the disruption of monolayers of cultured mammalian cells, such as CHO cells *in vitro*, and the *in vitro* virulence was abolished by the addition of CP inhibitor E64 (**Supplementary Figure S7B**). These results further support the premise that expression of EhArfX2^T28N^ inhibited transport of CPs to lysosomes from the trans-Golgi network and caused subsequent mistargeting of CPs to the culture medium. These results indicate that the transport of lysosomal enzymes including CPs is regulated by EhArfX2 and also suggest that EhArfX2^T28N^ expression may influence the pathogenesis of the parasite through CPs.

### Expression of EhArfX2^T28N^ Causes a Reduction in Abscess Formation

**Figure 4:**
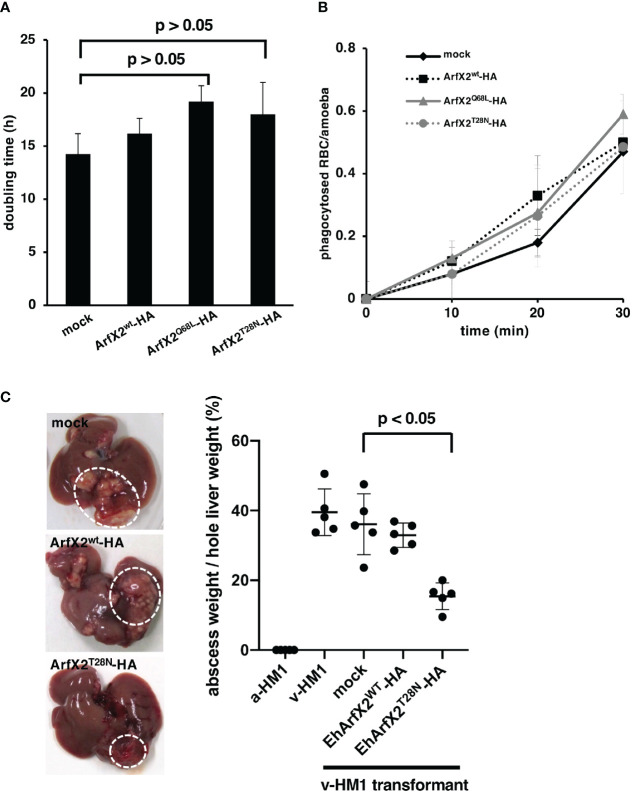
Effects of EhArfX2^WT^-HA, EhArfX2^Q68L^-HA, and EhArfX2^T28N^-HA expression on growth, erythrophagocytosis, and liver abscess formation. **(A)** The doubling time of EhArfX2^WT^-HA, EhArfX2^Q68L^-HA, EhArfX2^T28N^-HA, and mock control cells (n = 3). **(B)** Efficiency of phagocytosis of human erythrocytes. Amoeba transformant cells were incubated with human erythrocytes at the indicated times. Cells were fixed with paraformaldehyde, and the number of ingested erythrocytes were counted under the microscope (n = 30). **(C)** Amoebic liver abscess formation caused by infection with a-HM1, v-HM1, v-HM1 transformants expressing EhArfX2^WT^-HA, EhArfX2^T28N^-HA, and mock control cells. Six days after inoculation, hamsters were sacrificed and whole liver and separated liver abscesses were weighted (n = 5). Statistical significance was evaluated by the Student’s t-test.

To further investigate the causal connection between EhArfX2 expression and pathogenic activities, we next evaluated *in vitro* cell growth (**Figure 4A**) and ingestion of erythrocytes *via* trogocytosis or phagocytosis (Ralston et al., 2014; Somlata et al., 2017) (**Figure 4B**). The population doubling time of EhArfX2^WT^-, EhArfX2^Q68L^-, and EhArfX2^T28N^-expressing lines was unchanged when compared with the mock control (**Figure 4A**). In addition, erythrophagocytosis activity was also comparable among the strains (**Figure 4B**). We next evaluated the effect of EhArfX2^WT^ and the mutants on *in vivo* virulence, e.g., liver abscess formation, using the hamster liver abscess model. Based on the disturbed traffic of CPs observed in EhArfX2^T28N^-expressing transformant line (**Figure 3D**), it was expected that EhArfX2^T28N^-expressing lines may show a change in the ability of liver abscess formation. The transformant lines expressing EhArfX2^WT^ or dominant-negative EhArfX2^T28N^ were established using v-HM1 as a parental line and then injected into the hamster liver (**Figure 4C**). It should be noted that none of the transformants derived from a-HM1 was capable of forming liver abscesses in hamsters. Overexpression of EhArfX2^WT^-HA in v-HM1 did not affect the apparent *in vivo* virulence, i.e., liver abscess formation, when compared to mock control strain. Parental (non-transformant) v-HM1, mock transformant, and EhArfX2^WT^-HA-expressing transformant strains formed abscesses that occupied 39.5% ± 6.7%, 36% ± 8.7%, and 32.9% ± 3.4% of the whole liver weight, respectively. In contrast, EhArfX2^T28N^-HA-expressing strain formed significantly smaller abscesses, accounting for 15.4% ± 3.8% of the whole liver weight. These results indicate that expression of EhArfX2^T28N^, which caused impairment of lysosome biogenesis and regulation of CP trafficking, also resulted in a reduction in the ability of liver abscess formation despite comparable rate of growth and erythrophagocytosis *in vitro.* Overexpression of EhArfX2^WT^-HA upon v-HM1 genetic background did not have an additive effect on liver abscess formation.

### EhArfX2^T28N^ Caused a Defect in Nitrosative Stress Response

**Figure 5:**
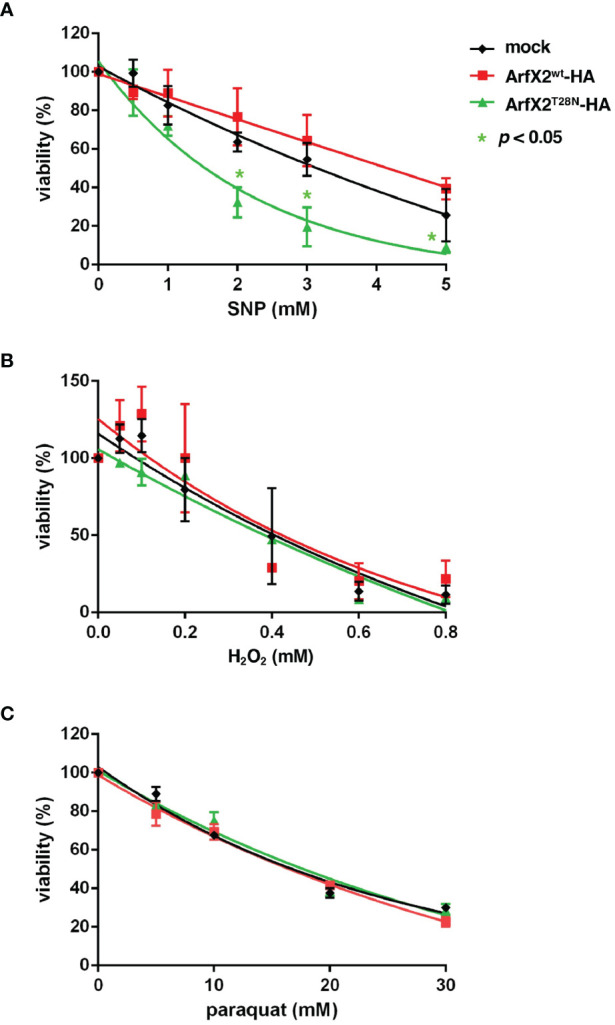
*In vitro* sensitivity to oxidative and nitrative stress of EhArfX2^WT^-HA and EhArfX2^T28N^-HA expression. Viability of trophozoites of EhArfX2^WT^-HA, EhArfX2^T28N^-HA, and mock transformants after 18-h treatment with varying concentrations of sodium nitroprusside (SNP) **(A)**, H_2_O_2_ **(B)**, or paraquat **(C)** is shown.

We speculated that EhArfX2^T28N^-expressing transformant has an additional defect other than oversecretion of CPs that explains less capacity for liver abscess formation. To this end, we hypothesized if EhArfX2^T28N^-expressing transformant has decreased resistance to environmental stresses because, during liver abscess formation, trophozoites are exposed to oxidative or nitrosative stresses, which are mainly produced by activated macrophages (Lin and Chadee, 1992; Dodson et al., 2013; Jeelani and Nozaki, 2016). The viability of EhArfX2^T28N^-HA- or EhArfX2^WT^-HA-expressing transformant that had been created from v-HM1 was evaluated under the exposure of nitrosative [sodium nitroprusside (SNP)] (Santi-Rocca et al., 2012) or oxidative (H_2_O_2_ and paraquat) stresses (Husain et al., 2012). Interestingly, EhArfX2^T28N^-HA-expressing line showed increased sensitivity to SNP when compared to EhArfX2^WT^-HA-expressing or mock transformant (**Figure 5A**). In contrast, sensitivity to H_2_O_2_ and paraquat was comparable to that of EhArfX2 WT-expressing and mock lines (**Figures 5B, C**). These results indicate that EhArfX2 is involved in the anti-nitrosative stress response.

## Discussion

### EhArfX2 Is Involved in Cysteine Protease Transport, Nitrosative Stress Response, and Amoebic Liver Abscess Formation

In this study, we have demonstrated for the first time that Arf GTPase plays important roles in intracellular trafficking of the major virulence-associated factor (CPs) and the defense against nitrosative stresses and is thus involved in liver abscess formation in animals. This is the first report on the involvement of Arf GTPases in the pathogenesis of *E. histolytica* and on the engagement of Arf GTPases in anti-nitrosative stress responses. It has been well established that Rab-regulated membrane trafficking (Nakada-Tsukui et al., 2005; Mitra et al., 2007; Saito-Nakano et al., 2007; Hanadate et al., 2016; Saito-Nakano et al., 2021), calcium and phosphoinositide signaling (Bharadwaj et al., 2017; Somlata et al., 2017; Nakada-Tsukui et al., 2019; Sharma et al., 2019; Babuta et al., 2020; Watanabe et al., 2020), and cytoskeletal regulation (Sahoo et al., 2004; Aslam et al., 2012; Mansuri et al., 2014; Babuta et al., 2018) play important roles in host cell recognition and ingestion (phago- and trogocytosis), secretion of virulence factors, and degradation of ingested materials in the lysosomes, trogosomes, and phagosomes. Thus, this study has underpinned Arf GTPase as a new player that regulates vesicular trafficking involved in the traffic of virulence factors, stress response, and thus *in vivo* virulence. The implications of this study should provide insights into multiple roles of membrane trafficking in diverse cellular functions in *E. histolytica.*

### Discovery of EhArfX2 as a Key Determinant of *In Vivo* Virulence by Comparative Transcriptomic Analysis of Virulent and Avirulent Isogenic *E. histolytica* Strains

It was striking that *EhArfX2* gene was exclusively identified among >100 *Rab*, 14 *Arf*/*Sar*, and other traffic-related genes including SNAREs and coatomer proteins (COPs) by transcriptomic comparisons of liver-passed v-HM1 and *in vitro* culture-attenuated a-HM1. These data underpin EhArfX2 to be a key determinant in liver abscess formation. Two studies of comparative transcriptomic analysis of attenuated and animal-passaged virulent strains with an identical genetic background were previously conducted (Santi-Rocca et al., 2008; Meyer et al., 2016). EhArfX2 was identified in neither of the studies. In the study by Santi-Rocca et al. (2008), 33 genes were found to be upregulated (>2-fold) in the animal-passed virulent HM-1 when compared to the attenuated avirulent strain. One of the genes encoded a parasite surface-associated protein, lysine and glutamic acid rich protein 1 (KERP1), and was further investigated, showing that KERP1 was involved in the adherence to the host cell and liver abscess development (Santi-Rocca et al., 2008). Meyer et al. (2016) identified 30 genes including tyrosine kinase and Myb family proteins as upregulated genes by comparing two isogenic clones: non-virulent HM-1:IMSS-A and virulent HM-1:IMSS-B clones. Among 30 genes, it was shown that gene silencing of one hypothetical protein EHI_127670 caused a decrease in CP secretion *in vitro* and the liver pathology *in vivo* when expression was induced in HM-1:IMSS-B (Meyer et al., 2016). In our study, the original strain we used was clonal and the genome was sequenced (Kawano-Sugaya et al., 2020), and we obtained virulent and attenuated lines derived from this clonal strain of HM-1:IMSS Cl6 for transcriptomic comparisons. The fact that EhArfX2 was not identified in the two previous studies (Santi-Rocca et al., 2008; Meyer et al., 2016) suggests the presence of multiple mechanisms for attenuation and virulence because no universal gene expression change, either upregulation or downregulation of a gene or a set of genes, was identified in three independent studies (**Table 1** and **Supplementary Table S2**). Note that genes encoding so-called classic virulence factors, such as Gal/GalNAc lectin, CPs, and amoebapores, were not differentially expressed, similar to the observations in two previous reports (Santi-Rocca et al., 2008; Meyer et al., 2016). The uniqueness and significance of the present study are that a novel EhArfX2 GTPase was identified to be involved in liver abscess development and nitrosative stress resistance (**Figure 5**).

### EhArfX2 Is Involved in Lysosome Trafficking From the Trans-Golgi Network and Lysosome Biogenesis

Arf/Sar GTPases are the key regulators of eukaryotic cell organization and membrane traffic together with Rab GTPases (Jackson and Bouvet, 2014). We identified 11 genes encoding EhArfs and three genes encoding EhSar1 in the *E. histolytica* genome (**Figure 1A**). N-terminal glycine residue, responsible for an N-terminal myristoylation and the insertion into the lipid bilayer, is conserved in EhArfX2, but the MxxE motif characteristic for Golgi-resident Arf1 is not present (**Figure 1D**). It has been shown that in human, the binding of ER-residing Arf1 to a membrane-localized SNARE in the cis-Golgi targeting is necessary for the ER–to–cis-Golgi traffic (Honda et al., 2005). We have demonstrated the trans-Golgi localization of EhArfX2 (**Figure 2F**), utilizing Golgi-resident luminal EhGalT and two SNAREs. The presence of Golgi-localized glycosylation enzymes was previously suggested by the bioinformatic approach in *Entamoeba* (Magnelli et al., 2008). In this study, we have shown that the small dot-like localization of one of the representative glycosylation enzymes from *E. histolytica*, EhGalT, responsible for the transfer of Gal, GalNAc, and GlcNAc to proteins, was closely associated with the ER (**Supplementary Figure S1C**), suggesting that amoebic glycosylation occurs at the region adjacent to the ER, similar to the case of the Golgi in plants, where the Golgi stacks are located in the vicinity of the ER exit site (Ito et al., 2018). EhGalT was not colocalized with EhArfX2 (**Figure 2B**), indicating that EhArfX2 does not localize to the EhGalT-positive Golgi membrane. SNAREs are essential for the fusion of transport vesicles with an acceptor membrane (Hong, 2005). The mechanisms of action and the subcellular localization of all SNAREs have been established in human, *Arabidopsis*, and *Saccharomyces* (Gupta and Brent Heath, 2002; Uemura et al., 2004; Hong, 2005; Kloepper et al., 2007). Phylogenetic analysis of 16 Q-SNAREs and nine R-SNAREs from *E. histolytica* predicted EhSed5 and EhYkt6 being associated with cis- and trans-Golgi, respectively (**Supplementary Figures S2A**, **S3A**). The short (15 aa) transmembrane region in EhSed5 well agrees with the premise of EhSed5 being a Golgi membrane resident (**Supplementary Table S4**). The Golgi-residing transmembrane proteins are typically short and likely due to the fact that the cholesterol and its analogs are less abundant in the Golgi apparatus than in the plasma membrane in humans (Bretscher and Munro, 1993). It is known that the transmembrane region of Sed5 orthologs is rich in phenylalanine, but its significance is not fully understood (Bretscher and Munro, 1993). Double prenylation of Ykt6 members at the C-terminus is essential for the membrane targeting of Ykt6 and the correct sorting of lysosomal hydrolases, cathepsin D and β-hexosaminidase, at the trans-Golgi in humans (Sakata et al., 2021) (**Supplementary Figure S3B**). It was shown that mutations of the C-terminus cysteine residues caused the missorting of lysosomal hydrolases at the trans-Golgi, leading to their missecretion into the extracellular space (Sakata et al., 2021). Taken together, these data imply the functional relevance of EhArfX2 and EhYkt6 in the trafficking from the trans-Golgi to the lysosomes in *E. histolytica* (**Figures 2D**, **3D**). Clearly distinct localization of EhSed5 and EhYkt6 is consistent with the premise that the Golgi membrane is functionally divided into cis- and trans-cisternae in *E. histolytica* (**Supplementary Figure S4C**), although the Golgi morphology was previously described as vacuole-like (Mazzuco et al., 1997).

### Dominant-Negative Effect of EhArfX2^T28N^

It was shown in human cells that Arf1^Q71L^ constitutively binds to GTP and inhibits the ER–to–Golgi and intra-Golgi transport *via* its dominant active effects (Dascher and Balch, 1994; Donaldson and Jackson, 2011). Expression of a GDP-bound inactive form of human Arf1 (Arf1^T31N^) failed to recruit coat protein I (COPI) coatomer to the membrane and inhibited the pre-Golgi transport (Dascher and Balch, 1994; Beck et al., 2009). Thus, the COPI uncoating reaction depends on GTP hydrolysis by Arf1, and both Arf mutants prevent the traffic from the Golgi (Tanigawa et al., 1993). In the present study, expression of either EhArfX2^Q68L^ or EhArfX2^T28N^ mutant showed positive or negative effects on lysosome biogenesis, respectively (**Figure 3A**). However, neither showed a strong dominant effect on cell growth and erythrophagocytosis activity (**Figures 4A, B**). Thus, the role of the GTPase cycle of EhArfX2 may differ from that of human Arf1, and the GTP-bound state may be required for the EhArfX2 function. The fact that lysosomal traffic was inhibited by the expression of EhArfX2^T28N^ mutant (**Figures 3D, E**) supported the hypothesis that GTP-bound state of EhArfX2 is required for the lysosomal fusion (**Figure 3A**). EhArfX2^T28N^ displayed reduced lysosome formation and abnormal accumulation of CP in the pre-lysosomal non-acidic compartment (**Figures 3D, E**). It remains to be investigated how CPs are transported from the trans-Golgi to lysosomes in an EhArfX2-dependent fashion.

Localization of overexpressed EhArfX2^WT^-HA to the cytosol and EhArfX2^T28N^-HA to the membrane fraction, demonstrated by subcellular fractionation and immunoblot analyses (**Figure 3C**), was unexpected because it was previously shown in human cells that wild-type Arf1–Arf5 were, when overexpressed, mainly localized to the Golgi and the amount of wild-type Arf1–Arf5 distributed to the cytosol also increased. Furthermore, inactive GDP-bound Arf1–Arf5 mutants in humans were also shown to be accumulated in the cytosol and formed a complex with Arf guanine nucleotide exchange factors (Arf GEFs) but failed to be activated at the Golgi membrane (Cohen and Donaldson, 2010). However, it has been recently shown that Arf-GDP on the Golgi membrane can recruit Arf GEF *via* an unidentified protease-sensitive receptor (Quilty et al., 2018). Arf is released from the Golgi membrane once GTP is hydrolyzed. It was speculated that the limited amount of the receptor controls the constitutive level of Arf-GTP on the Golgi membrane (Quilty et al., 2018). Identification of Arf GEF, responsible for GTP loading on EhArfX2, and an Arf GEF receptor on the trans-Golgi membrane may further clarify the detailed trafficking pathway from the trans-Golgi to lysosomes in *E. histolytica*. It is worth noting that seven candidates for Arf GEF, Sec7, are present in the *E. histolytica* genome (Pipaliya et al., 2019).

### EhArfX2 Is Involved in Anti-Nitrosative Stress Response During Liver Abscess Formation

*E. histolytica* CPs have been implicated for liver pathology because antisense inhibition of expression of CP-A5 reduced activity on formation of liver abscess in hamsters (Ankri et al., 1999). Thus, we expected that expression of EhArfX2^T28N^, which caused increased secretion of CP (**Figure 3D**), may also result in more pronounced hepatic abscesses *in vivo*. However, the results were counterintuitive: EhArfX2^T28N^ expression increased the *in vitro* CHO monolayer destruction (**Supplementary Figure S7A**), while it reduced the size of liver abscesses (**Figure 4C**). It is conceivable that *in vivo* virulence requires a tight regulation of the processing, posttranslational modifications (PTMs), and trafficking of EhCP-A5 at the trans-Golgi and lysosomes. However, the tight regulation of CP trafficking was presumably affected by overexpression of EhArfX2^T28N^-HA, leading to dysregulation and missecretion of the immature non-functional (or less active) CPs. Alternatively, unidentified factors, which are involved in, e.g., anti-nitrosative defense, are less efficiently transported in EhArfX2^T28N^ strain, leading to the reduction of *in vivo* virulence. The major virulent factor, EhCP-A5, is predicted to be glycosylated at Asn272 (Bruchhaus et al., 2003), and N-linked glycosylation on EhCP-A5 was verified by its inhibition by tunicamycin (Nakada-Tsukui et al., 2012). Thus, our findings, together with the implications by previous studies, suggest that the correct processing (i.e., a cleavage of the SP and the pro domain) and posttranslational modifications, including N-glycosylation, in the ER, the trans-Golgi, and lysosomes are impaired in EhArfX2^T28N^-expressing strain, and CP was missecreted into the extracellular milieu. It should be noted that the *in vitro* CP assay used in the present study (**Figure 3D** and **Supplementary Figure S7A**) does not distinguish precursor unprocessed (or partially processed) CPs and fully matured CPs because precursor CPs are also expected to be self-activated *in vitro*. Further investigations are needed to assess the pathological role of EhCP-A5 in tissue invasion by utilizing *ex vivo* model (Thibeaux et al., 2012). It was worth noting that it was shown that gene silencing of one hypothetical protein EHI_127670 caused a decrease in CP secretion *in vitro* and the liver pathology *in vivo* when the gene was overexpressed in the apparently highly virulent liver-passaged line, HM-1:IMSS B (Meyer et al., 2016). The underlying reason for this apparent discrepancy from our observation remains elusive. These findings indicate that the essentiality of EhArfX2 for the *in vivo* survival and the liver abscess is not attributable to its involvement in traffic and regulated secretion of CPs but likely management of stress in the hostile host environment during liver abscess formation. The importance of EhArfX2 on cell survival was supported, indirectly, by the repeated failure of gene silencing of *EhArfX2* (Morf et al., 2013; Nakada-Tsukui et al., 2018) in v-HM1 strain (data not shown). We have shown that EhArfX2 is involved in the defense against nitrosative, but not oxidative, stresses. To date, the defense mechanisms of anti-nitrosative stress, which is mainly caused by nitric oxide (NO) from macrophages, are not clearly understood. During development of amoebic liver abscess, exposure of NO from macrophages primarily causes nitrosylation of cysteine residues in parasite proteins (Lin and Chadee, 1992), inactivation of key metabolic enzymes, and ER fragmentation (Santi-Rocca et al., 2012). More than 100 proteins including CPs, alcohol dehydrogenase (ADH), and lectin heavy subunit were previously reported to be S-nitrosylated (Siman-Tov and Ankri, 2003; Hertz et al., 2014), including two Rab GTPases (EHI_108610 EhRab1A and EHI_177520 EhRabX11) and one Sar1 GTPase (EHI_075040 EhSar1c) (Hertz et al., 2014), indicating that the intracellular traffic between the ER to the Golgi is likely affected by NO.

In our comparative transcriptomic analysis, we also found that one of the Atg4 orthologs was upregulated in liver-passed v-HM1 (**Table 1**), suggesting the involvement of autophagy and possible cross talk with EhArfX2. Atg4 cleaves the ubiquitin-like Atg8 near the C-terminus glycine, allowing the conjugation of Atg8 with phosphatidylethanolamine (PE) to generate membrane-bound Atg8-PE for subsequent autophagosome formation (Kirisako et al., 2000; Kabeya et al., 2004). At the initial step of autophagosome formation, two different GTPases are required: TGN localized Arl1 and Ypt6 in *S. cerevisiae* (Yang and Rosenwald, 2016), which may partially explain a possible link between autophagy and post-TGN traffic and between autophagy and EhArfX2 in stress response. However, the specific role of EhAtg4 in trans-Golgi to lysosome traffic remains elusive. In mammalian cells, it has been demonstrated that damaged proteins by oxidative stress are degraded by the autophagy–lysosomal pathway under mild oxidative conditions (Dodson et al., 2013) because 26S proteasome is sensitive to oxidative stress (Ishii et al., 2005). Furthermore, it has been also demonstrated that the oxidative stress induces autophagy in mammalian cells (Scherz-Shouval et al., 2007). It has been also shown that under starvation conditions, cells produce reactive oxygen species (ROS), specifically H_2_O_2_, and inhibit Atg4 (Kirisako et al., 2000). Although it is not known if nitrosative stress also affects autophagy, damaged proteins by oxidative and nitrosative stresses may be degraded by lysosomal and autophagic degradation systems during host tissue invasion and liver abscess development.

## Data Availability Statement

The datasets presented in this study can be found in online repositories. The names of the repository/repositories and accession number(s) can be found below: https://www.ddbj.nig.ac.jp/, EhArfX1a (BR001420), EhArfX1b (BR001421), EhArfX2 (BR001422), EhArfX3 (BR001423), EhArfX4 (BR001424), EhArfX5 (BR001425), EhArfX6 (BR001426), EhArfX7 (BR001427), EhArfX8 (BR001428), EhArfX9 (BR001429), EhArfX10 (BR001430), EhSar1a (BR001431), EhSar1b (BR001432), EhSar1c (BR001433), EhSed5 (BR001434), EhSyn1A (BR001435), EhSyn1B (BR001436), EhSyn1C (BR001437), EhSynA (BR001438), EhSynB (BR001439), EhSynC (BR001440), EhSynD (BR001441), EhSynE (BR001442), EhSynF (BR001443), EhSynG (BR001444), EhSynH (BR001445), EhSynI (BR001446), EhSynJ (BR001447), EhVti11 (BR001448), EhVti12 (BR001449), EhYkt6 (BR001450), EhVampB (BR001451), EhVampD1 (BR001452), EhVampD2 (BR001453), EhVampD3 (BR001454), EhVampE (BR001455), EhVampF (BR001456), EhVampG (BR001457), EhVampH (BR001458).

## Ethics Statement

The animal study was reviewed and approved by the Institutional Animal Care and Use Committee (No. 211075) and conducted at the AAALAC-accredited National Institute of Infectious Diseases, Japan.

## Author Contributions

Conceptualization: YS-N, MT, WP, and TN. Investigation: YS-N, TM, and MT. Analyzed the data: YS-N, TM, and MT. Contributed analysis tools: CG and WP. Funding acquisition: YS-N and TN. Writing—original draft: YS-N, TM, MT, and TN. Writing—review and editing: YS-N, TM, MT, CG, WP, and TN. All authors contributed to the article and approved the submitted version.

## Funding

This research was partially funded by Grants-in-Aid for Scientific Research (C) (JP19K07531 to YS-N) and Grant-in-Aid for Scientific Research (B) (JP18H0265, JP21H02723) to TN from the Japan Society for the Promotion of Science, Grant for Research on Emerging and Re-emerging Infectious Diseases from Japan Agency for Medical Research and Development (AMED, JP20fk0108138 and JP20fk0108139 to YS-N and TN), and a Grant for Science and Technology Research Partnership for Sustainable Development (SATREPS) from AMED and Japan International Cooperation Agency (JICA) (JP20jm0110022) to TN.

## Conflict of Interest

The authors declare that the research was conducted in the absence of any commercial or financial relationships that could be construed as a potential conflict of interest.

## Publisher’s Note

All claims expressed in this article are solely those of the authors and do not necessarily represent those of their affiliated organizations, or those of the publisher, the editors and the reviewers. Any product that may be evaluated in this article, or claim that may be made by its manufacturer, is not guaranteed or endorsed by the publisher.
