# Corrigendum: ArfX2 GTPase Regulates Trafficking From the Trans-Golgi to Lysosomes and Is Necessary for Liver Abscess Formation in the Protozoan Parasite *Entamoeba histolytica*

**DOI:** 10.3389/fcimb.2022.856477

**Published:** 2022-03-18

**Authors:** Yumiko Saito-Nakano, Takashi Makiuchi, Mami Tochikura, Carol A. Gilchrist, William A. Petri, Tomoyoshi Nozaki

**Affiliations:** ^1^Department of Parasitology, National Institute of Infectious Diseases, Shinjuku-ku, Tokyo, Japan; ^2^Department of Infectious Diseases, Tokai University School of Medicine, Isehara, Kanagawa, Japan; ^3^Department of Medicine, University of Virginia, Charlottesville, VA, United States; ^4^Graduate School of Medicine, The University of Tokyo, Bunkyo, Tokyo, Japan

**Keywords:** *Entamoeba histolytica*, liver abscess, membrane traffic, Arf GTPase, stress resistance

In the original article, there was a mistake in the Supplemental Table numbers for **Supplemental Tables 1**–**4** as published. This was because the **Supplemental Figure legend file** was designated as **Supplemental Table 1**.

The authors apologize for this error and state that this does not change the scientific conclusions of the article in any way. The original article has been updated.

## Publisher’s Note

All claims expressed in this article are solely those of the authors and do not necessarily represent those of their affiliated organizations, or those of the publisher, the editors and the reviewers. Any product that may be evaluated in this article, or claim that may be made by its manufacturer, is not guaranteed or endorsed by the publisher.

