# Supplementary figures and images for: ArfX2 GTPase Regulates Trafficking From the Trans-Golgi to Lysosomes and Is Necessary for Liver Abscess Formation in the Protozoan Parasite Entamoeba histolytica
Source: Front Cell Infect Microbiol. 2021 Dec 17;11:794152. doi: 10.3389/fcimb.2021.794152 (PMC8719317; doi:10.3389/fcimb.2021.794152)

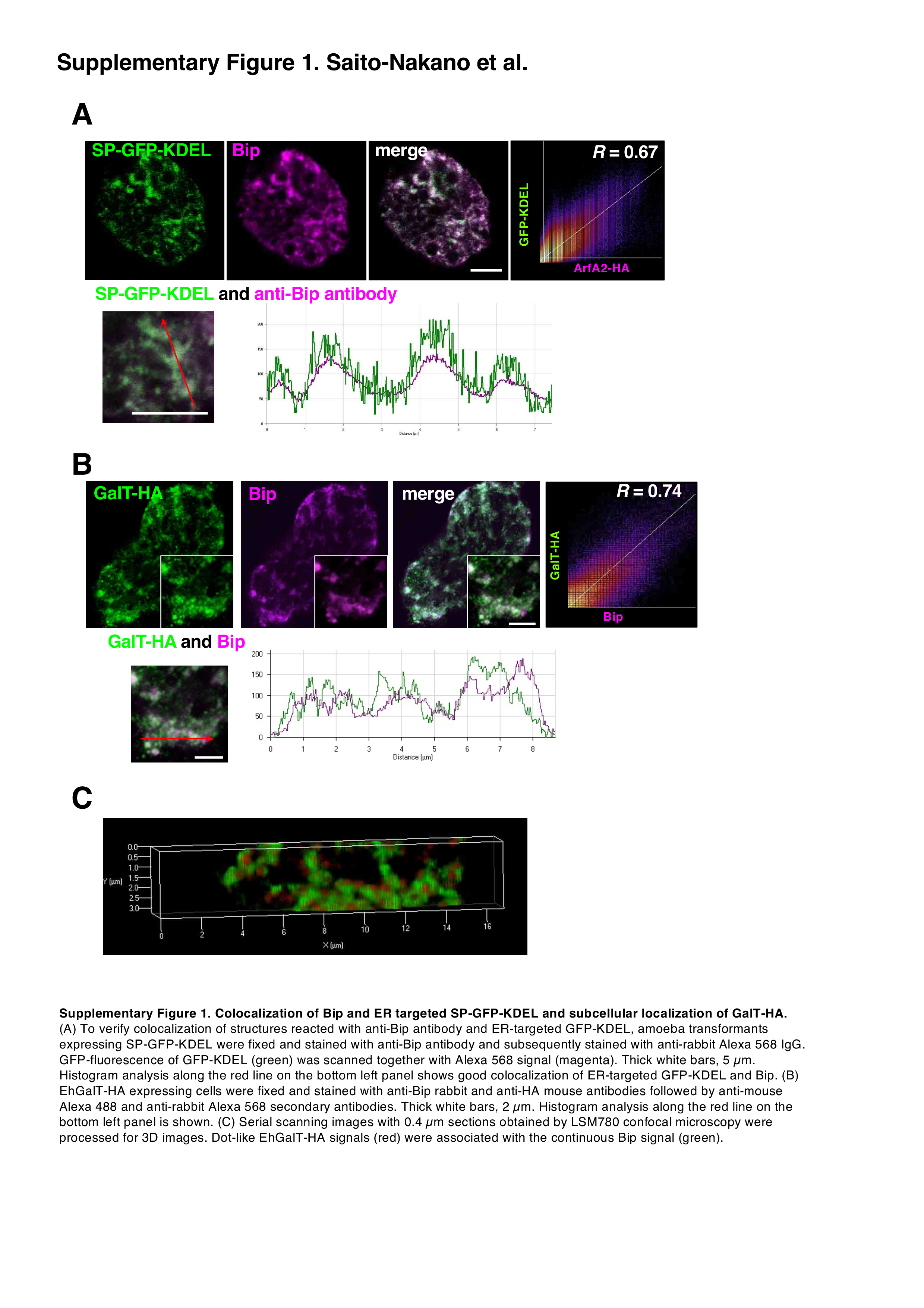

Supplement: Supplementary file 1 [file Image_1.tiff]

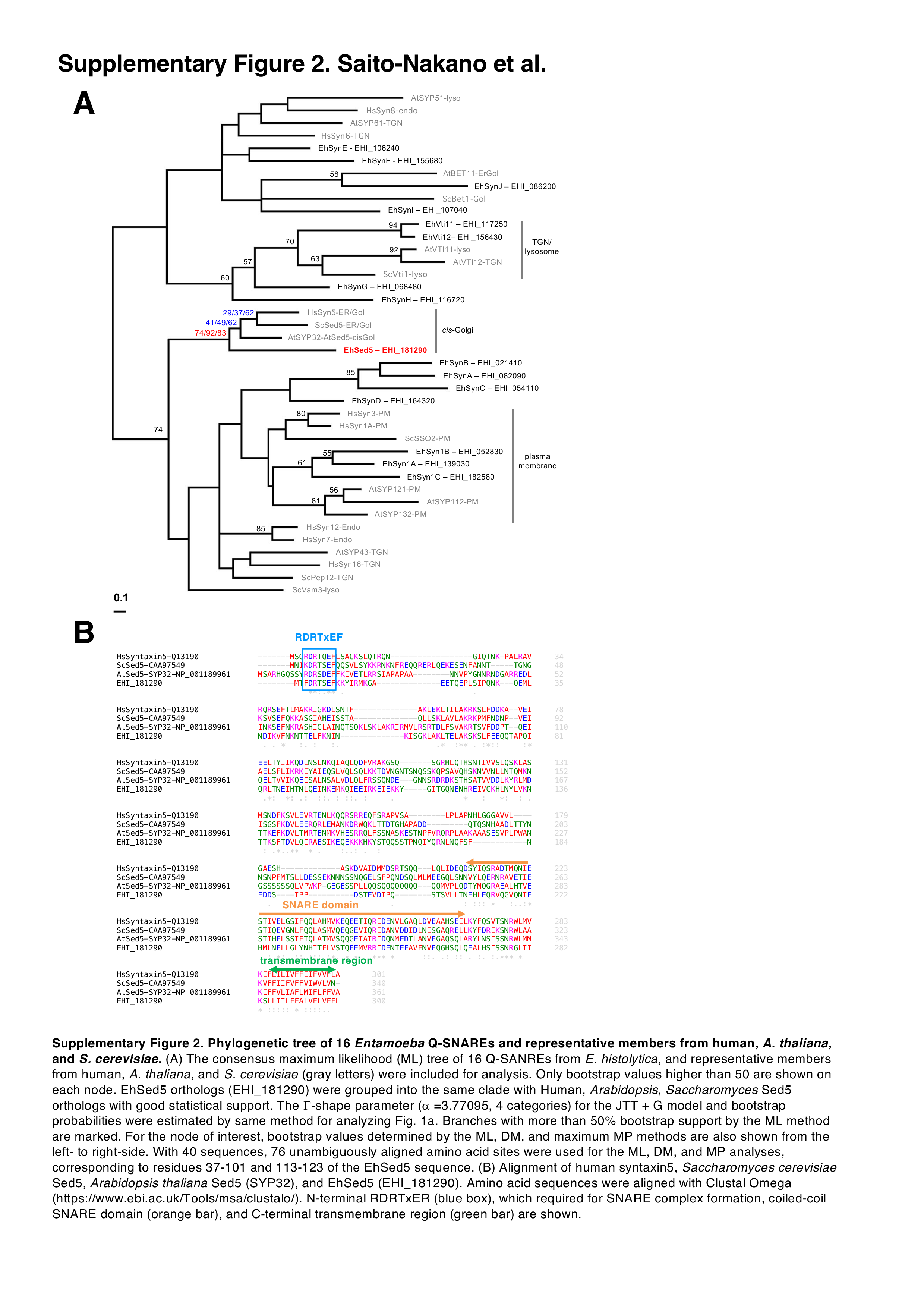

Supplement: Supplementary file 2 [file Image_2.tiff]

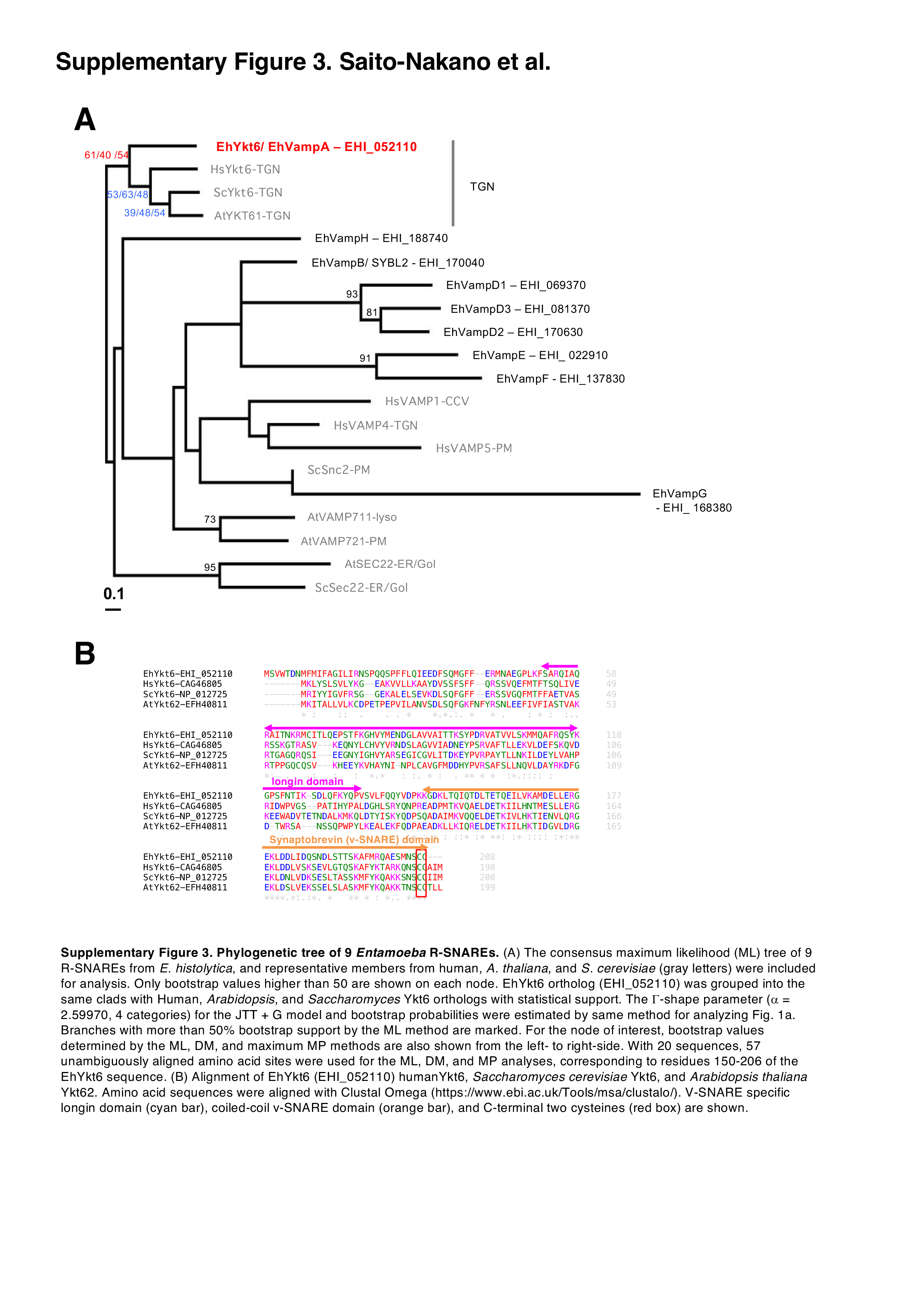

Supplement: Supplementary file 3 [file Image_3.tiff]

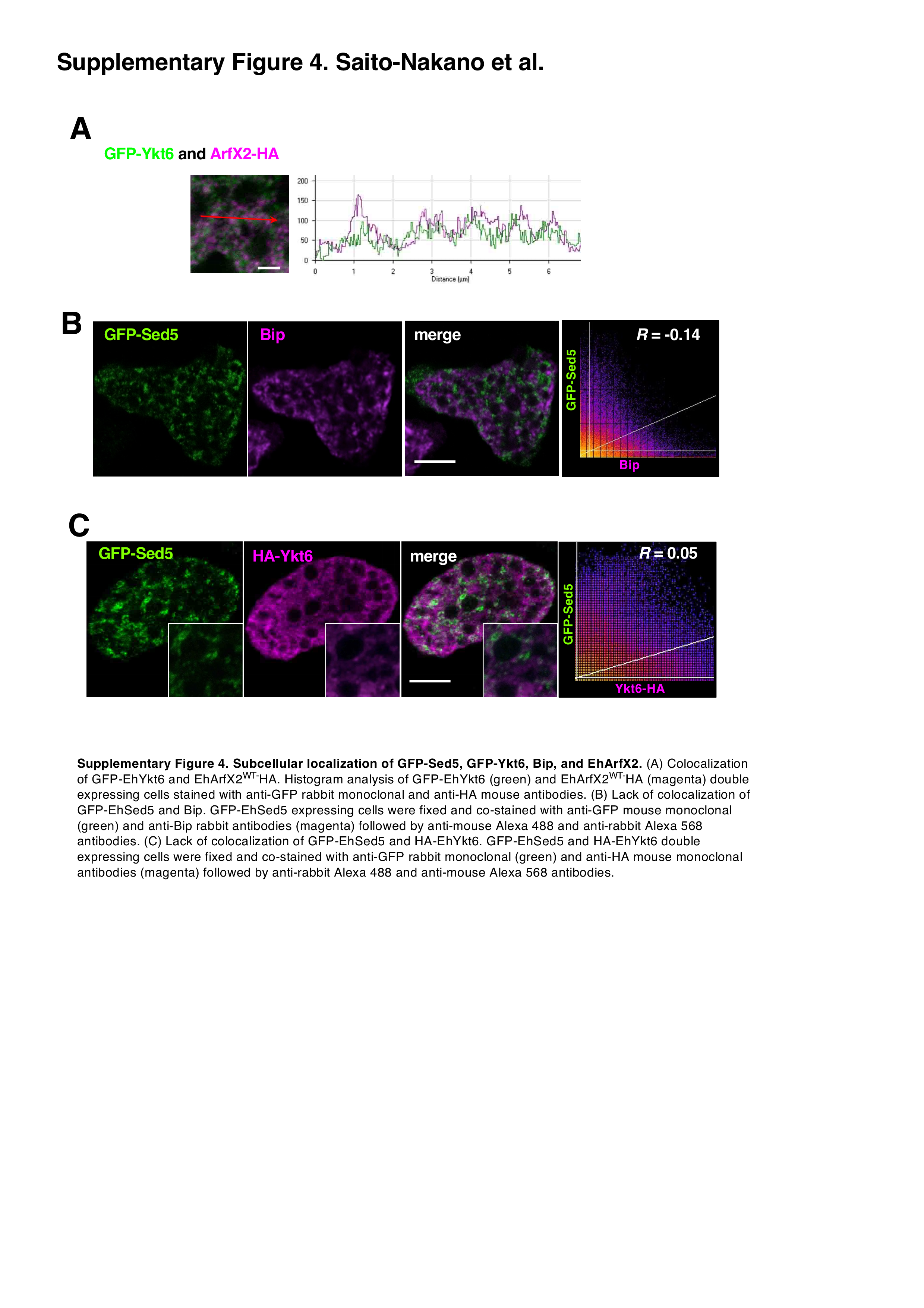

Supplement: Supplementary file 4 [file Image_4.tiff]

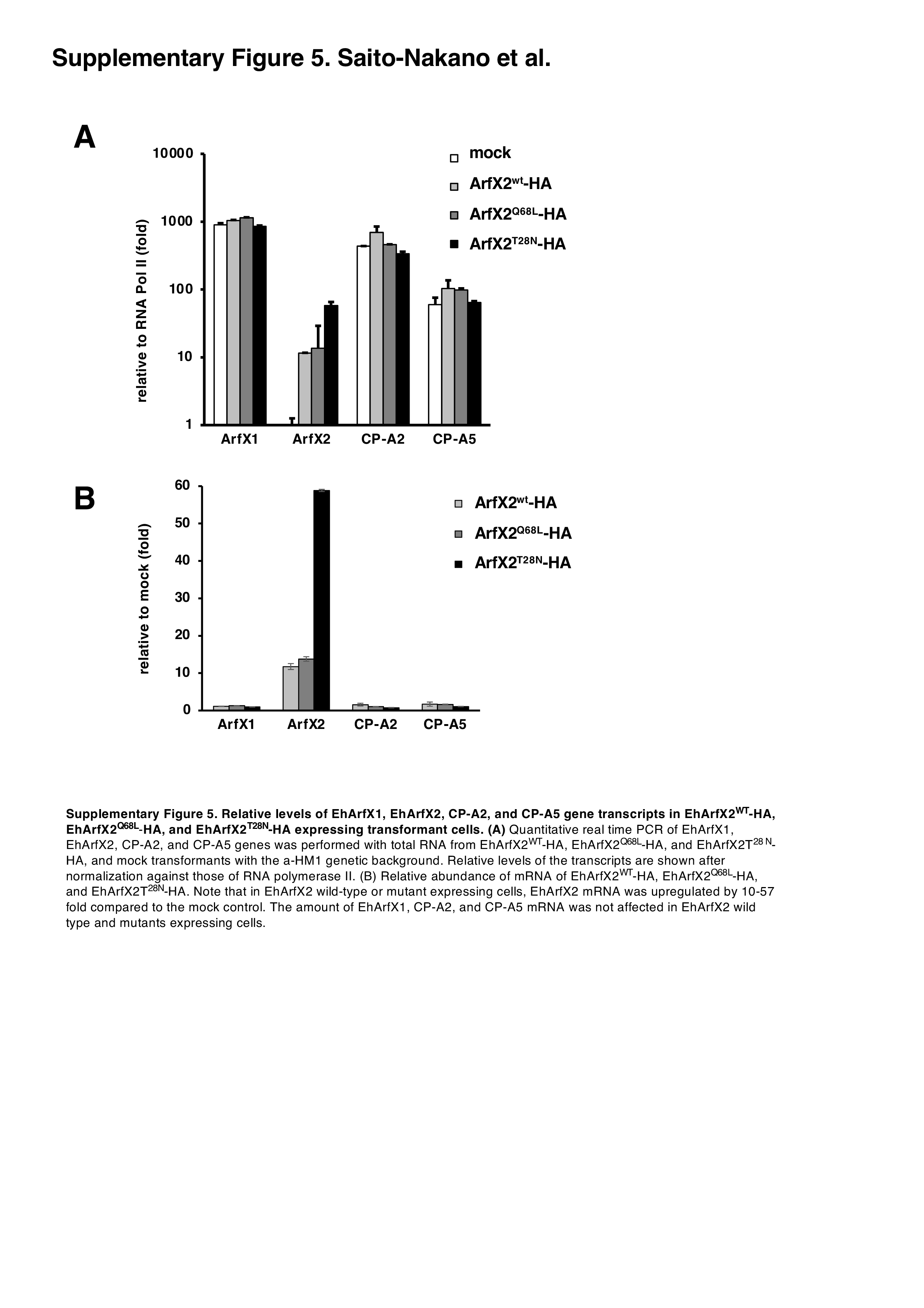

Supplement: Supplementary file 5 [file Image_5.tiff]

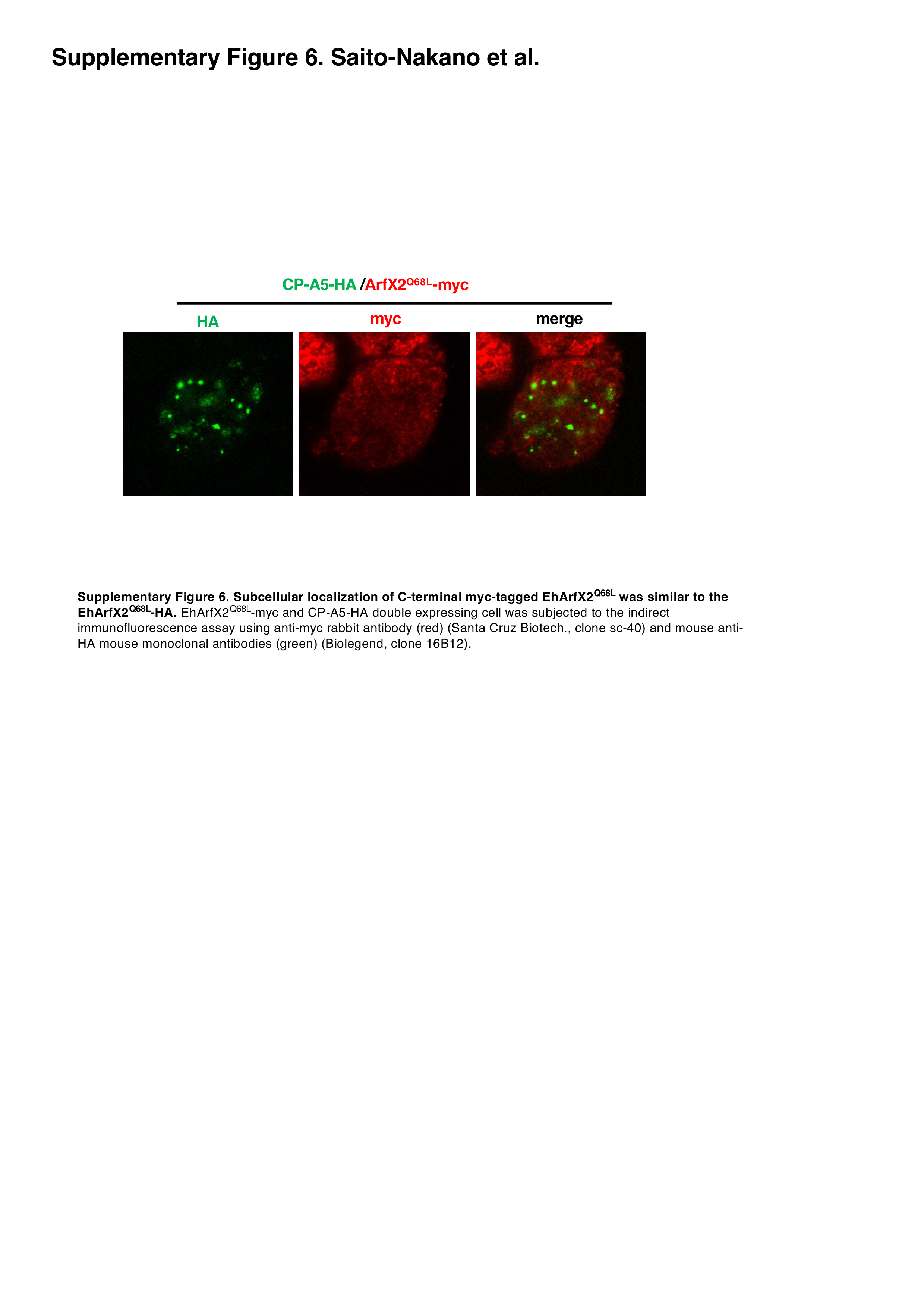

Supplement: Supplementary file 6 [file Image_6.tiff]

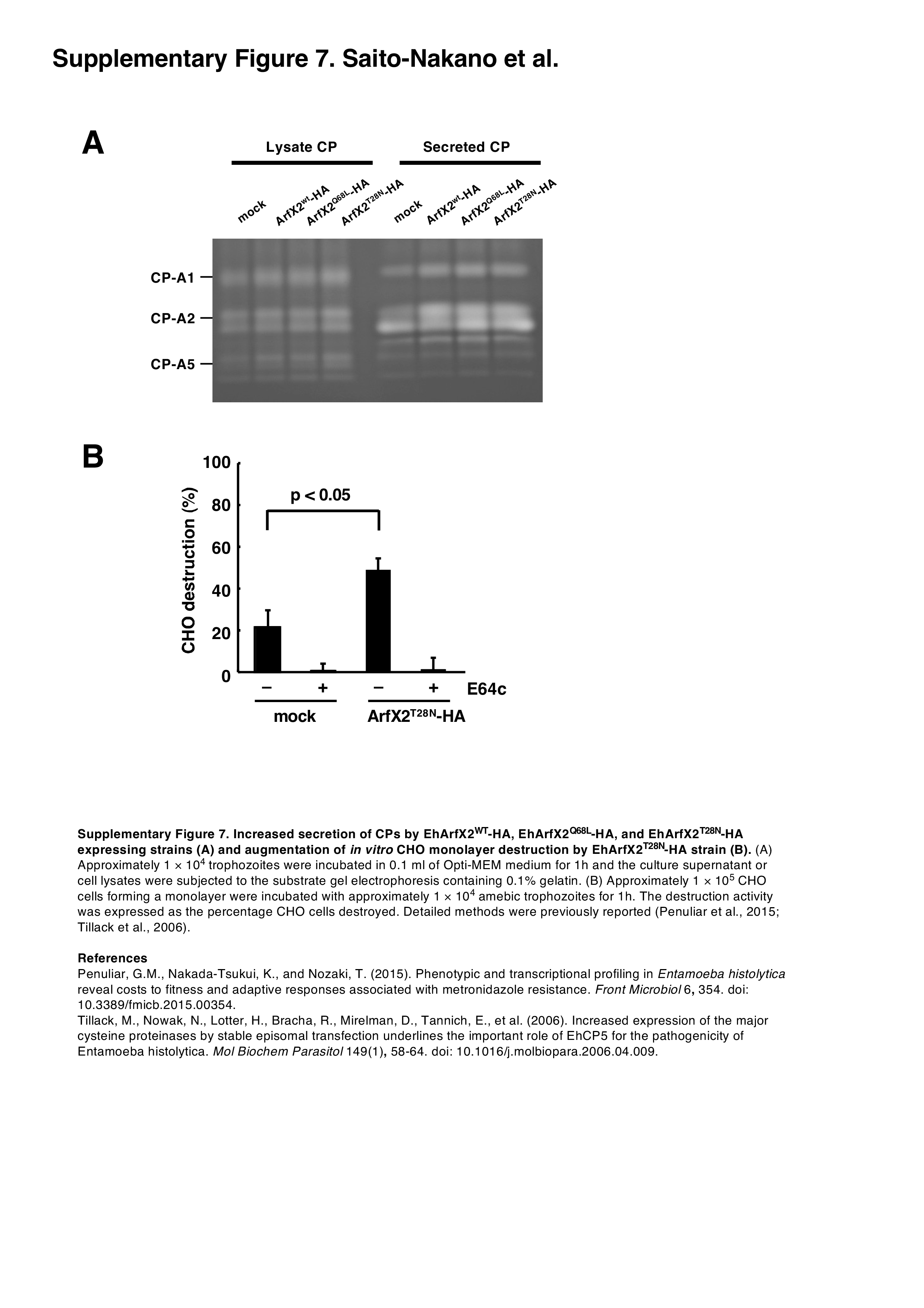

Supplement: Supplementary file 7 [file Image_7.tiff]
