# Supplementary material for: ArfX2 GTPase Regulates Trafficking From the Trans-Golgi to Lysosomes and Is Necessary for Liver Abscess Formation in the Protozoan Parasite Entamoeba histolytica
Source: Front Cell Infect Microbiol. 2021 Dec 17;11:794152. doi: 10.3389/fcimb.2021.794152 (PMC8719317; doi:10.3389/fcimb.2021.794152)
Supplement: Supplementary file 8 [file Presentation_1.pdf]

Supplementary Material

Supplementary Figure 1. Colocalization of Bip and ER targeted SP-GFP-KDEL and subcellular localization of GalT-HA. (A) To verify colocalization of structures reacted with anti-Bip antibody and ER-targeted GFP-KDEL, amoeba transformants expressing SP-GFP-KDEL were fixed and stained with anti-Bip antibody and subsequently stained with anti-rabbit Alexa 568 IgG. GFP-fluorescence of GFP-KDEL (green) was scanned together with Alexa 568 signal (magenta). Thick white bars, 5 μm. Histogram analysis along the red line on the bottom left panel shows good colocalization of ER-targeted GFP-KDEL and Bip. (B) EhGalT-HA expressing cells were fixed and stained with anti-Bip rabbit and anti-HA mouse antibodies followed by anti-mouse Alexa 488 and anti-rabbit Alexa 568 secondary antibodies. Thick white bars, 2 μm. Histogram analysis along the red line on the bottom left panel is shown. (C) Serial scanning images with 0.4 μm sections obtained by LSM780 confocal microscopy were processed for 3D images. Dot-like EhGalT-HA signals (red) were associated with the continuous Bip signal (green).

Supplementary Figure 2. Phylogenetic tree of 16 *Entamoeba* Q-SNAREs and representative members from human, *A. thaliana*, and *S. cerevisiae*. (A) The consensus maximum likelihood (ML) tree of 16 Q-SANREs from *E. histolytica*, and representative members from human, *A. thaliana*, and *S. cerevisiae* (gray letters) were included for analysis. Only bootstrap values higher than 50 are shown on each node. EhSed5 orthologs (EHI_181290) were grouped into the same clade with Human, *Arabidopsis*, *Saccharomyces* Sed5 orthologs with good statistical support. The Γ-shape parameter (α =3.77095, 4 categories) for the JTT + G model and bootstrap probabilities were estimated by same method for analyzing Fig. 1a. Branches with more than 50% bootstrap support by the ML method are marked. For the node of interest, bootstrap values determined by the ML, DM, and maximum MP methods are also shown from the left- to right-side. With 40 sequences, 76 unambiguously aligned amino acid sites were used for the ML, DM, and MP analyses, corresponding to residues 37-101 and 113-123 of the EhSed5 sequence. (B) Alignment of human syntaxin5, *Saccharomyces cerevisiae* Sed5, *Arabidopsis thaliana* Sed5 (SYP32), and EhSed5 (EHI_181290). Amino acid sequences were aligned with Clustal Omega (https://www.ebi.ac.uk/Tools/msa/clustalo/). N-terminal RDRTxER (blue box), which required for SNARE complex formation, coiled-coil SNARE domain (orange bar), and C-terminal transmembrane region (green bar) are shown.

Supplementary Figure 3. Phylogenetic tree of 9 *Entamoeba* R-SNAREs. (A) The consensus maximum likelihood (ML) tree of 9 R-SNAREs from *E. histolytica*, and representative members from human, *A. thaliana*, and *S. cerevisiae* (gray letters) were included for analysis. Only bootstrap values higher than 50 are shown on each node. EhYkt6 ortholog (EHI_052110) was grouped into the same clads with Human, *Arabidopsis*, and *Saccharomyces* Ykt6 orthologs with statistical support. The Γ-shape parameter (α = 2.59970, 4 categories) for the JTT + G model and bootstrap probabilities were estimated by same method for analyzing Fig. 1a. Branches with more than 50% bootstrap support by the ML method are marked. For the node of interest, bootstrap values determined by the ML, DM, and maximum MP methods are also shown from the left- to right-side. With 20 sequences, 57 unambiguously aligned amino acid sites were used for the ML, DM, and MP analyses, corresponding to residues 150-206 of the EhYkt6 sequence. (B) Alignment of EhYkt6 (EHI_052110) humanYkt6, *Saccharomyces cerevisiae* Ykt6, and *Arabidopsis thaliana* Ykt62. Amino acid sequences were aligned with Clustal Omega (https://www.ebi.ac.uk/Tools/msa/clustalo/). V-SNARE

specific longin domain (cyan bar), coiled-coil v-SNARE domain (orange bar), and C-terminal two cysteines (red box) are shown.

Supplementary Figure 4. Subcellular localization of GFP-Sed5, GFP-Ykt6, Bip, and EhArfX2. (A) Colocalization of GFP-EhYkt6 and EhArfX2^{wt}-HA. Histogram analysis of GFP-EhYkt6 (green) and EhArfX2^{wt}-HA (magenta) double expressing cells stained with anti-GFP rabbit monoclonal and anti-HA mouse antibodies. (B) Lack of colocalization of GFP-EhSed5 and Bip. GFP-EhSed5 expressing cells were fixed and co-stained with anti-GFP mouse monoclonal (green) and anti-Bip rabbit antibodies (magenta) followed by anti-mouse Alexa 488 and anti-rabbit Alexa 568 antibodies. (C) Lack of colocalization of GFP-EhSed5 and HA-EhYkt6. GFP-EhSed5 and HA-EhYkt6 double expressing cells were fixed and co-stained with anti-GFP rabbit monoclonal (green) and anti-HA mouse monoclonal antibodies (magenta) followed by anti-rabbit Alexa 488 and anti-mouse Alexa 568 antibodies.

Supplementary Figure 5. Relative levels of EhArfX1, EhArfX2, CP-A2, and CP-A5 gene transcripts in EhArfX2^{WT}-HA, EhArfX2^{Q68L}-HA, and EhArfX2^{T28N}-HA expressing transformant cells. (A) Quantitative real time PCR of EhArfX1, EhArfX2, CP-A2, and CP-A5 genes was performed with total RNA from EhArfX2^{WT}-HA, EhArfX2^{Q68L}-HA, and EhArfX2T^{28N}-HA, and mock transformants with the a-HM1 genetic background. Relative levels of the transcripts are shown after normalization against those of RNA polymerase II. (B) Relative abundance of mRNA of EhArfX2^{WT}-HA, EhArfX2^{Q68L}-HA, and EhArfX2T^{28N}-HA. Note that in EhArfX2 wild-type or mutant expressing cells, EhArfX2 mRNA was upregulated by 10-57 fold compared to the mock control. The amount of EhArfX1, CP-A2, and CP-A5 mRNA was not affected in EhArfX2 wild type and mutants expressing cells.

Supplementary Figure 6. Subcellular localization of C-terminal myc-tagged EhArfX2^{Q68L} was similar to the EhArfX2^{Q68L}-HA. EhArfX2^{Q68L}-myc and CP-A5-HA double expressing cell was subjected to the indirect immunofluorescence assay using anti-myc rabbit antibody (red) (Santa Cruz Biotech., clone sc-40) and mouse anti-HA mouse monoclonal antibodies (green) (Biolegend, clone 16B12).

Supplementary Figure 7. Increased secretion of CPs by EhArfX2^{WT}-HA, EhArfX2^{Q68L}-HA, and EhArfX2^{T28N}-HA expressing strains (A) and augmentation of *in vitro* CHO monolayer destruction by EhArfX2^{T28N}-HA strain (B). (A) Approximately 1×10^4 trophozoites were incubated in 0.1 ml of Opti-MEM medium for 1h and the culture supernatant or cell lysates were subjected to the substrate gel electrophoresis containing 0.1% gelatin. (B) Approximately 1×10^5 CHO cells forming a monolayer were incubated with approximately 1×10^4 amebic trophozoites for 1h. The destruction activity was expressed as the percentage CHO cells destroyed. Detailed methods were previously reported (Penuliar et al., 2015; Tillack et al., 2006).

References

Penuliar, G.M., Nakada-Tsukui, K., and Nozaki, T. (2015). Phenotypic and transcriptional profiling in *Entamoeba histolytica* reveal costs to fitness and adaptive responses associated with metronidazole resistance. *Front Microbiol* 6, 354. doi: 10.3389/fmicb.2015.00354.

Tillack, M., Nowak, N., Lotter, H., Bracha, R., Mirelman, D., Tannich, E., et al. (2006). Increased expression of the major cysteine proteinases by stable episomal transfection underlines the important

role of EhCP5 for the pathogenicity of *Entamoeba histolytica*. *Mol Biochem Parasitol* 149(1)**,** 58-64. doi: 10.1016/j.molbiopara.2006.04.009.
